# CLIC4 regulates apical exocytosis and renal tube luminogenesis through retromer- and actin-mediated endocytic trafficking

**DOI:** 10.1038/ncomms10412

**Published:** 2016-01-20

**Authors:** Szu-Yi Chou, Kuo-Shun Hsu, Wataru Otsu, Ya-Chu Hsu, Yun-Cin Luo, Celine Yeh, Syed S. Shehab, Jie Chen, Vincent Shieh, Guo-an He, Michael B. Marean, Diane Felsen, Aihao Ding, Dix P. Poppas, Jen-Zen Chuang, Ching-Hwa Sung

**Affiliations:** 1 Department of Ophthalmology, Weill Cornell Medical College, 1300 York Avenue, New York City, New York 10065, USA; 2 Institute for Pediatric Urology, Department of Urology, Weill Cornell Medical College, 1300 York Avenue, New York City, New York 10065, USA; 3 Department of Microbiology and Immunology, Weill Cornell Medical College, 1300 York Avenue, New York City, New York 10065, USA; 4 Department of Cell and Developmental Biology, Weill Cornell Medical College, 1300 York Avenue, New York City, New York 10065, USA

## Abstract

Chloride intracellular channel 4 (CLIC4) is a mammalian homologue of EXC-4 whose mutation is associated with cystic excretory canals in nematodes. Here we show that CLIC4-null mouse embryos exhibit impaired renal tubulogenesis. In both developing and developed kidneys, CLIC4 is specifically enriched in the proximal tubule epithelial cells, in which CLIC4 is important for luminal delivery, microvillus morphogenesis, and endolysosomal biogenesis. Adult CLIC4-null proximal tubules display aberrant dilation. In MDCK 3D cultures, CLIC4 is expressed on early endosome, recycling endosome and apical transport carriers before reaching its steady-state apical membrane localization in mature lumen. CLIC4 suppression causes impaired apical vesicle coalescence and central lumen formation, a phenotype that can be rescued by Rab8 and Cdc42. Furthermore, we show that retromer- and branched actin-mediated trafficking on early endosome regulates apical delivery during early luminogenesis. CLIC4 selectively modulates retromer-mediated apical transport by negatively regulating the formation of branched actin on early endosomes.

## Abstract (web summary)

Chloride intracellular channel (CLIC) 4 is an ion channel, localized in the cytoplasm, and first identified as an actin binding protein. Here, Chou *et al.* knockout CLIC4 in mice and observe tubulogenesis and renal proximal tubule dilation defects, which is caused by irregular actin and endosomal trafficking.

## Graphical abstract

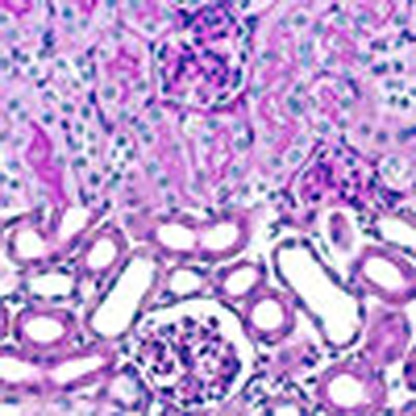

Studies carried out in Madin-Darby canine kidney (MDCK) three-dimensional (3D) ‘cyst' culture have greatly advanced our knowledge about the early phase of epithelial tubulogenesis (i.e., luminogenesis). They support a model in which the nascent lumen is formed *de novo* by coalescence of apical vesicles onto a specialized cell-cell contact (or apical membrane initiation site (AMIS)) followed by successive apical exocytoses through a Rab11a-Rab8-Cdc42-mediated pathway^1^,^2^,^3^. Interfering with any molecule important for the apical exocytic pathway uniformly leads to a multilumen phenotype^3^,^4^,^5^. While the role of apical exocytosis in *de novo* lumen formation has been established, the mechanism by which apical trafficking is regulated in a spatial-temporal fashion remains largely elusive^6^. Furthermore, despite its high relevance to human diseases, the molecular basis governing epithelial tube size/shape control and its relationship to the initial lumen formation are largely unexplored.

The excretory canal of *Caenorhabditis elegans*, the functional equivalent of the urinary system in mammals, is a single-celled tubule formed by coalescence of apical vesicles of the excretory cell^7^. The exc-4 gene cloned from the mutant worms that form a cystic canal encodes the mammalian homologue of chloride intracellular channel (CLIC)^8^. CLIC proteins are a family of six members (CLIC1-CLIC6)^9^. CLIC was named because of the intracellular microsomal localization of the founding member CLIC5B/p64 and its ability to generate an outwardly rectifying chloride channel activity when overexpressed heterologously^10^. Paradoxically, biochemical and structural analyses show CLIC4 is a cytosolic protein^11^,^12^. CLIC4 has recently been linked with vessel lumen formation in cultures, and angiogenesis in an oxygen-induced retinopathy model through a poorly understood mechanism^13^,^14^. CLIC4 was previously isolated in an actin-containing protein complex from brain lysates^15^. Actin plays pivotal roles in substructural organization, organelle biogenesis and vesicular trafficking^16^,^17^. The functional relevance of CLIC4 in actin-mediated cellular processes is elusive.

Endosomal compartments are where exocytic and endocytic pathways intersect^6^,^18^. Early endosome (EE), a pleomorphic organelle, contains an EEA1^+^ vacuolar domain and Rab11a^+^ tubular domains^19^,^20^,^21^. Rab11a^+^ vesicles budded from EE tubules coalesce to form the recycling endosome (RE), a way station for subsequent surface targeting^20^. On EE, proteins destined for degradation are sorted into intraluminal vesicles of the late endosome (LE) that later fuse with the lysosome^22^. Emerging studies have highlighted that retromer-regulated endocytic trafficking sorts cargoes from EE to several subcellular sites, including plasma membrane (PM), LE and *trans*-Golgi network (TGN)^23^,^24^. While the branched actin network plays a critical role in endosomal trafficking, how actin dynamics is regulated on the endosomal compartments remains poorly understood.

Here, we investigate CLIC4's role in renal tubulogenesis at the organogenic, cellular and molecular levels by using a novel CLIC4-knockout (KO) mouse model^25^, with CLIC4-knockdown (KD) MDCK 3D culture. The adult CLIC4-KO mice exhibited dilated renal proximal tubules (PTs). Developmental studies show these mice have tubulogenic defects accompanied by abnormal apical cargo delivery, endolysosomal genesis and microvillus (MV) morphogenesis. These phenotypes can be recapitulated in the MDCK 3D cysts and a PT epithelial cell line LLC-PK1. Our mechanistic studies suggest that CLIC4 is important for apical exocytosis during the early stage of luminogenesis, which results from its ability to regulate retromer-mediated transport by negatively modulating the branched actin formation on EE.

## Results

### CLIC4 distribution and function in developing kidney

**Figure 1:**
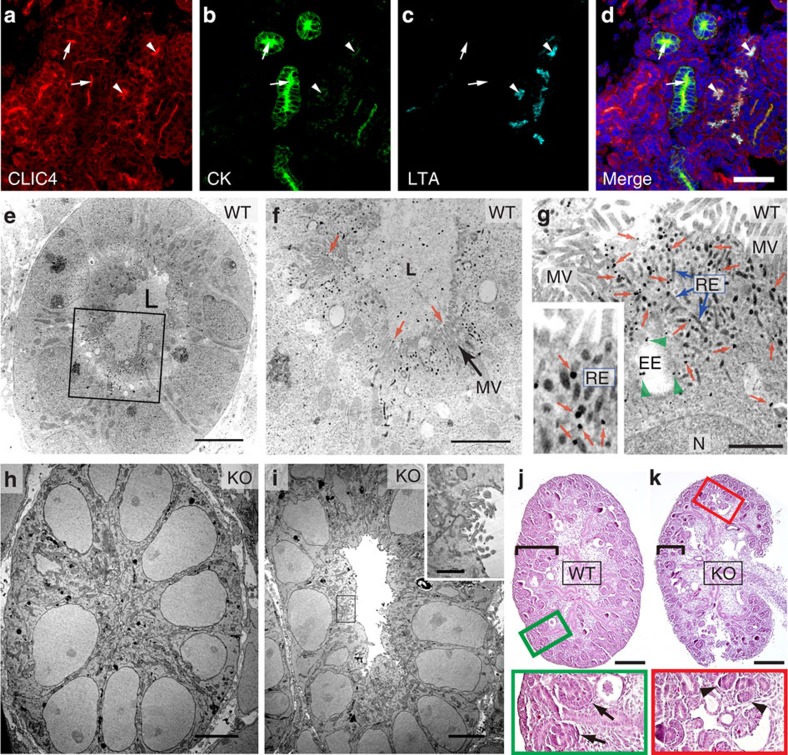
Expression of CLIC4 in embryonic kidney and characterization of CLIC4-KO metanephros. (**a**-**d**) E15 WT metanephros labelled for CLIC4, cytokeratin (CK) and LTA. DAPI: blue. Arrows and arrowheads point to the lumens of cytokeratin and LTA-labelled tubules, respectively. (**e**-**i**) Electron micrographs of pre-PT cells of WT (**e**-**g**) and CLIC4-KO (**h**-**i**) E15 metanephros labelled with CLIC4 immunogolds. The metanephros lumen (L) in WT is filled with electron-dense ECM. (**f**,**g**) Enlarged views of the boxed area in (**e**). CLIC4 immunogolds in MV (red arrows in **f**), EE (green arrowheads in **g**) and RE (blue arrows in **g**). The inset highlights the CLIC4 immunogolds (red arrows) on electron-dense RE tubules. N: nuclei. (**h**) A mutant PT without detectable central lumen. (**i**) A hypomorphic mutant PT containing an electron-dense ECM-devoid lumen. The inset shows the enlarged view of the boxed luminal surface demonstrating the presence of residual CLIC4 immunogolds on the short, irregular MV-like processes. (**j**,**k**) PAS staining of longitudinally sectioned E15 metanephros of WT (**j**) and CLIC4-KO (**k**) mice. Black brackets indicate the cortical areas. Enlarged views of boxed areas highlight the primitive tubules (arrows) and condensed mesenchyme clusters (arrowheads) found in the WT and KO, respectively. Scale bars, 50 μm (**d**); 5 μm (**e**,**h**,**i**); 2 μm (**f**); 1 μm (**g**, inset of **i**); 200 μm (**j**,**k**).

Rodent renal nephrons begin to develop embryonically, continue differentiation postnatally and reach maturation at ∼3 weeks of age^26^. Using a CLIC4-specific antibody (Supplementary Note 1 and Supplementary Fig. 1a,b), we showed that in E15 mouse embryonic kidney (i.e., metanephros) CLIC4 was concentrated in the luminal sides of almost all recognizable primitive tubules. These include the cytokeratin-expressing ureteric buds, and *Lotus tetragonolobus* agglutinin (LTA)-labelled pre-PTs (Fig. 1a-d and Supplementary Fig. 1a). As the kidney developed, CLIC4 became enriched exclusively in the LTA-labelled PT cells, and this nephron-specific expression remained throughout adulthood (Supplementary Fig. 1a for postnatal day 0 (PN 0); Supplementary Fig. 1c for adult). CLIC4 was also detected in glomerular and peritubular capillary endothelial cells, as previously reported^27^. At the subcellular level, CLIC4 was primarily enriched in the apical PM, which was spatially distinguishable from the subluminal Rab11a-labelled RE (Supplementary Fig. 1d).

To investigate CLIC4's role in renal tubular development, we systematically examined its ultrastructural distribution and the morphological defect of CLIC4-KO mice beginning in embryonic development. Using a (pre-embedding) immunoelectronic microscopic (EM) protocol that simultaneously preserves both antigenic epitopes and membrane structures, we showed that in wild-type (WT) E15 pre-PT cells, CLIC4 immunogolds were abundant in the apical cytoplasmic structures previously characterized as EE and RE (electron-dense tubules)^28^,^29^ (Fig. 1e-g). CLIC4 immunogolds were also detected in lumen surrounding rudimentary microvilli as well as inside the lumen. Negligible immunoreactivity was detected in the CLIC4-KO metanephros, validating the immunospecificity. Interestingly, many pre-PT like cells in the mutant metanephros did not display an open lumen (Fig. 1h). However, in the ones that did, their cavities did not contain electron-dense extracellular matrix (ECM), nor were they surrounded by MV (Fig. 1i). Close inspection of the latter indicated hypomorphism; sparse CLIC4 immunogolds were detected on the residual MV-like processes (Fig. 1i, inset).

At the light microscopic level, compared to WT, CLIC4-KO metanephros had thinner cortex (Fig. 1k versus j, Supplementary Fig. 1e), which contained fewer detectable regular-shaped tubules but more condensed mesenchyme clusters (Fig. 1j,k). Furthermore, while LTA (that labels glycoproteins) and apical membrane protein megalin^30^ predominantly labelled the central lumens of WT pre-PTs, such enrichment was reduced in the mutants (Supplementary Fig. 1f). These results collectively suggested that CLIC4 is important for the central lumen formation and MV morphogenesis during embryonic renal tubule development.

### Defects in newborn CLIC4-KO mice

**Figure 2:**
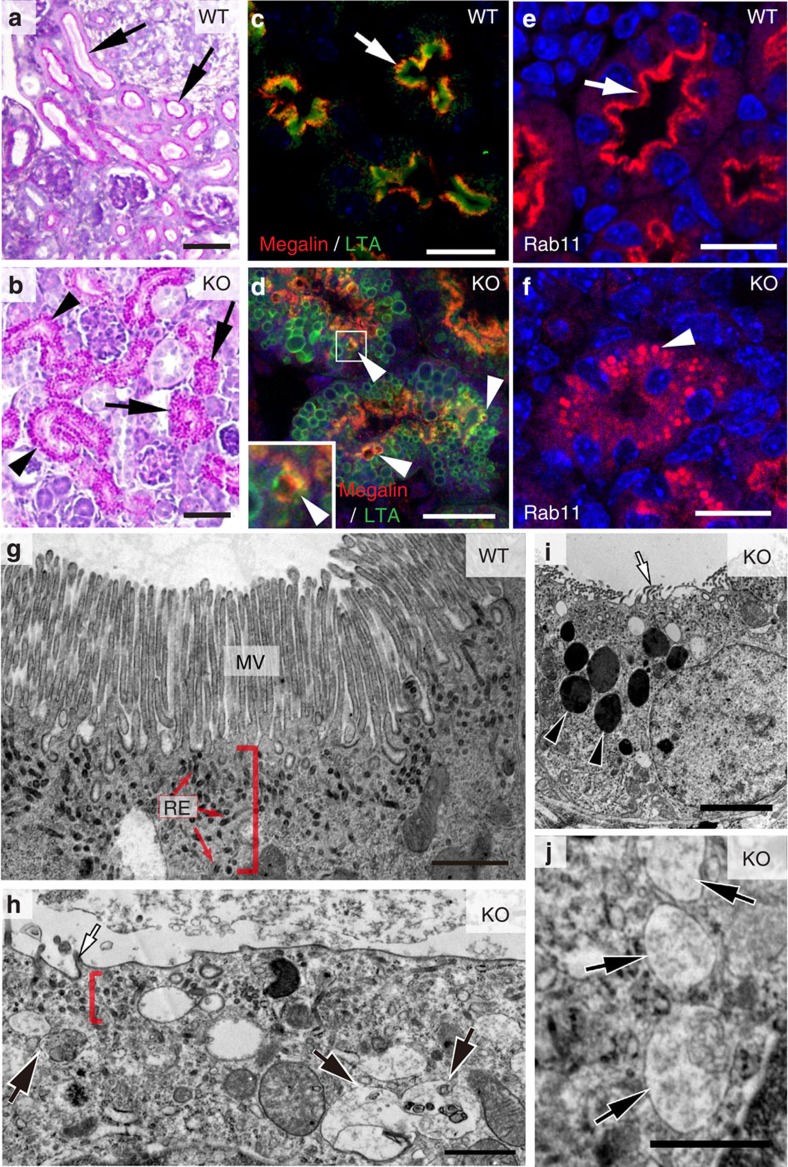
Lumen formation and maturation defect of CLIC4-KO developing PT. (**a**,**b**) PAS-stained renal cortical sections of PN0 WT (**a**) and CLIC4-KO (**b**) mice. Arrows in (**a**) point to the PTs displaying intense PAS+ luminal PM signals. Arrows and arrowheads in (**b**) point to the cell clusters and the primitive tubes, respectively; both of them contained PAS-positive granules. (**c**,**d**) LTA and megalin staining of PTs in PN0 WT(**c**) and CLIC4-KO (**d**) mice. Prominent luminal LTA and subluminal megalin signals (arrow) were seen in the WT. Mutant PTs displayed abundant LTA-labelled vacuoles; some contained mislocalized megalin (arrowheads). The inset shows the magnified view of the box area. (**e**,**f**) Immunolabelling of Rab11a in PN0 WT (**e**) and KO (**f**) PTs. An arrow points to the (sub)luminal lining pattern of Rab11a staining in WT. An arrowhead in (**f**) points to the granular/vacuolar staining pattern of the remaining Rab11a in mutant PT. (**g**-**j**) Representative electron micrographs of PN0 PTs in WT (**g**) and CLIC4-KO (**h-j**) mice. Red brackets indicate the apical cytoplasmic regions containing RE (red arrows), which are electron-dense tubules. Mutant PT cells did not develop typical MVs (white arrow, **h**,**i**), but contained unusually high numbers of LE-, autophagosome- (black arrows in **h**,**j**) and lysosome- (black arrowheads in **i**) like structures. Scale bars, 50 μm (**a**,**b**); 20 μm (**c-f**); 1 μm (**g**,**h**,**j**); 3 μm (**i**).

As development progressed, PN0 mutant kidneys were able to develop appreciable numbers of cortical tubules, indicating that the tubulogenic defect was partially overcome. However, while periodic acid–Schiff (PAS; stained glycoproteins) highlighted the luminal PM in the WT tubules, intense PAS staining was found on the cytoplasmic vacuoles in mutant PTs instead (Fig. 2a,b and Supplementary Fig. 2a). LTA and megalin staining were also displaced into the intracellular vacuoles from their typical MV/subapical locations (Fig. 2c,d). On the other hand, the apical and basolateral PM expression of NHE3 and Na^+^/K^+^–ATPase, respectively, was not detectably changed in the mutant PTs (Supplementary Fig. 2b,c). Together, these results suggested that CLIC4 is important for apical delivery, but not overall polarity.

Ultrastructural analyses showed that the mutant PT remained to be unable to develop proper MV (Fig. 2h versus g). Remarkably, the characteristic subapical zone containing EE and RE was also not evident in the mutant; the electron-dense apical RE tubules were difficult to find. On the other hand, the mutant had prominently increased numbers of late endosomal compartments, including the multivesicule-containing LE, electron-dense lysosome and their intermediates (Fig. 2g-j). In agreement with these findings, LTA-labelled PTs (but not other nephron segments) had increased LAMP1 (a lysosome marker; Supplementary Fig. 2d) and reduced Rab11a; the residual Rab11a signal was found in the intracellular vacuoles instead (Fig. 2f versus e). These results collectively indicate that CLIC4 is important for both luminogenesis and endolysosomal biogenesis during the early stage of renal tubulogenesis.

### Adult CLIC4-KO mice develop dilated PTs

**Figure 3:**
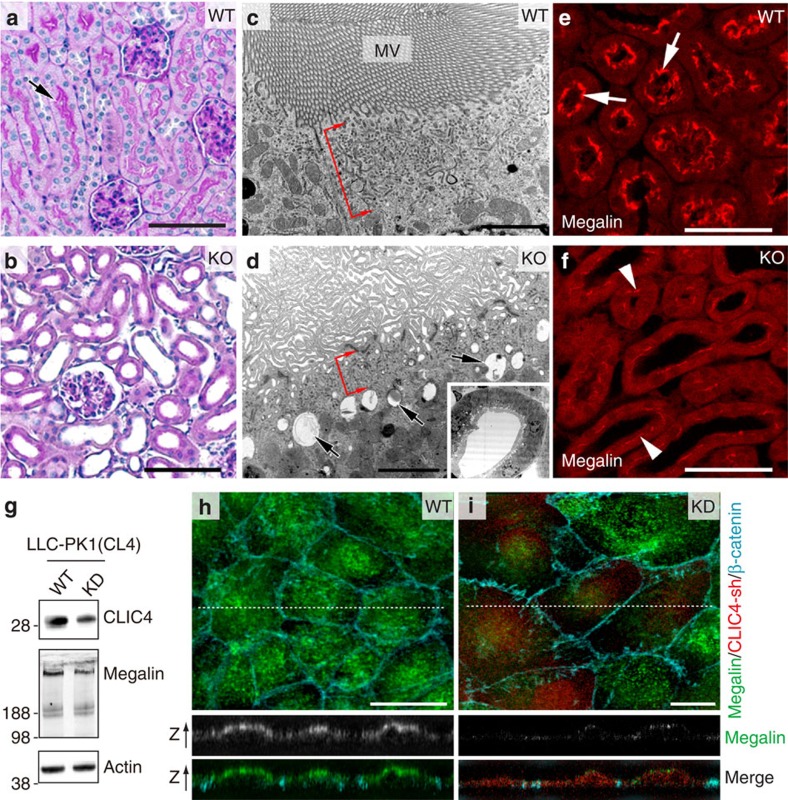
Adult CLIC4-KO PTs have dilated lumens and abnormal MV morphogenesis. (**a**,**b**) PAS staining of 3-week-old WT (**a**) and CLIC4-KO (**b**) renal cortical sections. An arrow in (**a**) points to strong PAS-labelled brush borders facing a PT lumen. (**c**,**d**) EM analysis of 3-week-old WT (**c**) and CLIC4-KO (**d**) mouse PT. Red brackets indicate the RE-containing apical cytoplasmic regions. Arrows in (**d**) point to the large vacuoles in the mutant PT. Inset in (**d**) shows a low-magnification view of a dilated PT that failed to develop a typical MV-lining lumen. (**e**,**f**) Megalin immunostaining of adult WT (**e**) and mutant (**f**) PTs. Megalin had a lumen outlining pattern (arrows) in WT. Mutant PTs displayed weak megalin signals (arrowheads). (**g**) Immunoblots of lysates of LLC-PK1(CL4) cells without (WT) or with (KD) doxycycline-induced CLIC4-shRNA expression. (**h**,**i**) LLC-PK1(CL4) cells, without (**h**) or with (**i**) expression of CLIC4-shRNA/RFP, stained with megalin and β-catenin. Representative confocal images of x-y (upper panel) and x-z (two lower panels; arrows point to the apical side of cells) views are shown. Note that in (**i**) the ‘red' CLIC4-shRNA-expressing cells had selectively reduced megalin signal. Scale bars, 100 μm (**a**,**b**); 2 μm (**c**,**d**); 50 μm (**e**,**f**); 10 μm (**h**,**i**).

CLIC4-KO mice had a lower birth rate than WT; in addition, some of the mice that were born died before they reached maturity. A small fraction (7 out of 50) of those mice examined displayed severe nephric histopathology (Supplementary Fig. 3a-c); we did not further analyse these mice in this paper. The rest of the surviving 3-week-old mutant mice had no gross histopathology in the renal medulla. However, almost all of them had abnormally dilated PTs (Fig. 3b versus a and Supplementary Fig. 3d). The mutant PTs did not display PAS-labelled ‘brush borders' (Fig. 3b versus a), which was confirmed by EM. Unlike WT, which exhibited densely packed long MV arrays (Fig. 3c), mutant PT cells had only sparse and disorganized membrane protrusions (Fig. 3d).

The adult mutant PT cells continued to have fewer REs and more lysosomes/vacuoles (Fig. 3d). At this stage, the apical labelling of megalin (Fig. 3e,f) and NHE3 (Supplementary Fig. 3e) was barely detectable. In fact, megalin reduction could be detected by immunoblotting as early as in PN0 kidney lysates (Supplementary Fig. 3f). Thus, CLIC4-null PTs appear to have dysregulated lysosomal degradation function.

To corroborate the *in vivo* studies, we generated and examined stable clones of the PT-derived cell line LLC-PK1(CL4)^31^ that expressed doxycycline-induced CLIC4-shRNA/RFP(Fig. 3g). As predicted^32^, endogenous megalin labelling was predominantly found on the apical side of untreated cells (Fig. 3h). By contrast, doxycycline treatment caused a drastic decrease of megalin signal in CLIC4-KD (indicated by RFP signal) cells, both by immunoblotting (Fig. 3g) and by staining (Fig. 3i). Furthermore, the density of both ezrin-labelled MV and Rab11a-labelled RE (Supplementary Fig. 3g,h) was markedly reduced in CLIC4-KD LLC-PK1(CL4) cells.

### CLIC4 associates with apical exocytosis during luminogenesis

**Figure 4:**
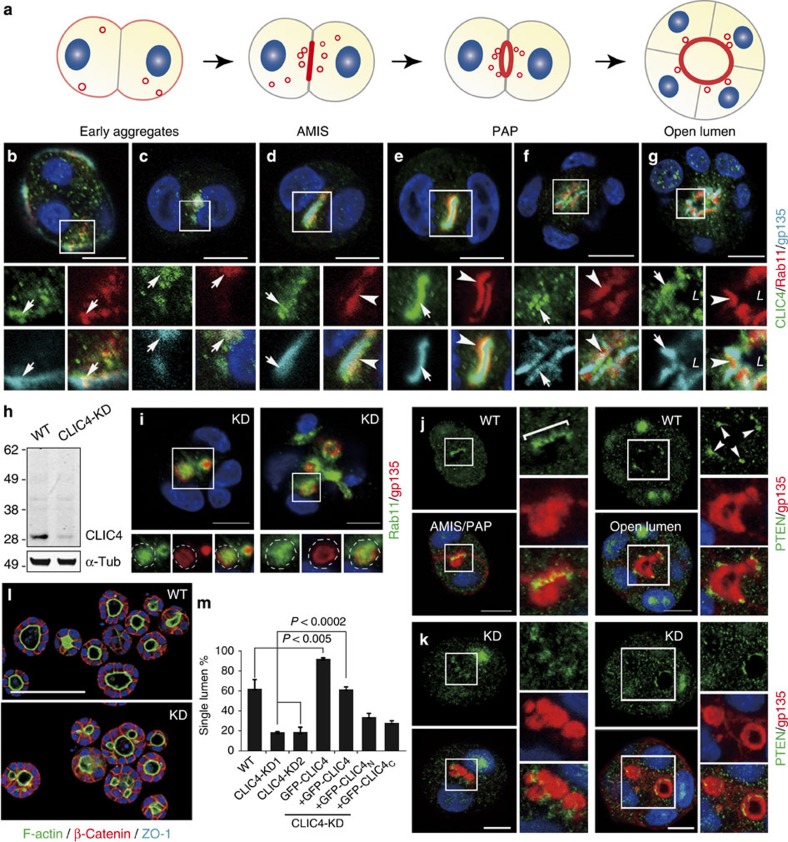
Expression and functional analyses of CLIC4 in MDCK luminogenesis. (**a**) A model illustrating the stepwise *de novo* lumen formation in a MDCK 3D cyst. Red and gray represent the membranes containing apical and basolateral molecules, respectively. Blue: nuclei. (**b-g**) The expression pattern of endogenous CLIC4 and gp135 in cysts formed by MDCK cells stably expressing mCherry-Rab11a. Images were taken from 12 h-2 day (early aggregates to PAP) and 3 days (open lumen) cultures. Arrows in (**b**,**c**) point to overlapping signals of CLIC4, gp135 and Rab11a. In more developed cysts (**d**-**g**), CLIC4, gp135 (arrows) and Rab11a (arrowheads) are spatially resolvable. L: lumen. (**h**) Immunoblots of lysates obtained from MDCK (WT) cells and a representative CLIC4-KD stable line. A single ∼28-kDa band of CLIC4 was specifically detected in WT MDCK lysates. CLIC4, but not the control protein. α-Tubulin (α-tub) was drastically diminished in the CLIC4-KD lysates. (**i**) Two representative examples of CLIC4-KD (2-day) cysts immunolabelled for gp135 and Rab11a. Enlarged views of the boxed areas show the accumulation of these two molecules (Pearson's coefficient=0.64±0.05; *n*=6) in the ectopic lumens/vacuoles (dash lines). (**j**,**k**) Immunolabelling of PTEN and gp135 in WT (**j**) and CLIC4-KD (**k**) cysts of different stages (1-2-day). Enlarged views of the boxed areas are also shown. In WT, PTEN was first enriched at AMIS/PAP (bracket) and then TJ (arrowheads). Similar enrichment was absent from the KD cysts. (**l**) Representative images of WT and CLIC4-KD (3-day) cysts labelled for F-actin, β-catenin, and ZO-1. (**m**) The percentages of the (3-day) cysts containing a signal lumen. Error bars represent standard deviation; *P* value by *t*-test; *n*>380; 3 repeats. Scale bars, 10 μm (**b**-**g**,**i**-**k**); 100 μm (**l**).

Next, we turned to *in vitro* cultures to investigate the mechanistic role of CLIC4 in luminogenesis. We utilized MDCK 3D cyst culture because it has been widely used to model early-phase renal tubulogenesis, despite the uncertainty regarding the nephron segment from which MDCK cells were derived. Previous studies showed that MDCK cells undergo a cell polarity reversal ∼12 h after culturing in Matrigel^3^. At the two-cell stage, the apical marker gp135 first appears on the cell surface in contact with ECM, and then Rab11a-bearing vesicles^3^ (Fig. 4a). Rab11a recruits additional exocytic components (e.g., Rab8, Cdc42) onto the apical vesicles *en route* to AMIS. Repeated fusions at the AMIS transform it into pre-apical patches (PAP) and then into an open luminal surface.

During the course of MDCK cyst maturation, CLIC4 was first found on the cell periphery, with gp135^+^/Rab11a^+^ apical vesicles (Fig. 4b,c), and then concentrated near the gp135-enriched AMIS juxtaposing Rab11a-labelled REs (Fig. 4d-g). The spatial-temporal association of CLIC4 with gp135 and Rab11 could be faithfully recapitulated by the stably expressed GFP-CLIC4 (Supplementary Fig. 4a,b), which was also evident in the MV-like protrusions in 6-day-old cysts (Supplementary Fig. 4c).

### CLIC4 is involved in apical vesicular coalescence

Several MDCK stable lines with ∼85% reduction of CLIC4 were generated (Fig. 4h shows an example). In the cysts formed by CLIC4-KD cells, apical proteins (gp135, aPKC, Rab11a) and tight junction (TJ) proteins (ZO-1, Par3) often failed to reach the predicted AMIS/luminal sites and nascent lumens; instead, they were retained in the ectopic vacuoles/lumens (Fig. 4i and Supplementary Fig. 4d,e). Unlike in WT cysts, where Rab11a and gp135 were largely separate from each other as PAP emerged (Fig. 4e-g), these two molecules had extensive overlap in the ectopic lumens of CLIC4-KD cysts (Fig. 4i), a sign of compromised apical cargo delivery. Consistent with this, an apically secreted protein gp80/clusterin, normally concentrated in the central lumen, was also found inside the ectopic lumens of the mutant cysts (Supplementary Fig. 4f). The basolateral marker β-catenin (Fig. 4l), however, distributed normally in CLIC4-KD cysts. Thus, CLIC4 is required for apical vesicle coalescence but not the overall apical-basolateral polarity.

Previous studies showed that targeting of Phosphatase and Tensin Homolog (PTEN) to the pre-AMIS is critical for the local enrichment of PI(4,5)P2, which serves as a hub for subsequent vesicle docking/fusion and luminal PM growth^2^,^33^. In WT cysts, PTEN was first concentrated in AMIS, and then TJ (Fig. 4j); however, such specific enrichment was not seen in the mutant (Fig. 4k). Consistent with the notion that AMIS targeting of PTEN is critical for luminogenesis, a significantly smaller fraction of 3-day CLIC4-KD cysts formed a single lumen compared to that of WT (Fig. 4l,m). Conversely, an increased percentage of cysts that stably expressed GFP-CLIC4 (∼4-fold over endogenous CLIC4) contained a single lumen (Fig. 4m). The multilumen phenotype of CLIC4-KD cells could be rescued by adding back the GFP fusion of full-length CLIC4 (aa 1-253), but not by two CLIC4 truncated variants (e.g., CLIC4_N_ contains aa 1-107, CLIC4_c_ contains aa 96-253) (Fig. 4l,m and Supplementary Fig. 5a,b). Concordantly, both CLIC4 truncates were unable to target to the luminal PM as could the full-length protein. GFP-CLIC4_N_ predominantly localized to ZO-1-labelled TJs and GFP-CLIC4_c_ had a diffuse cytoplasmic and nuclear distribution (Supplementary Fig. 5b). Thus, the full-length CLIC4 sequence is required for its proper targeting and luminogenic function.

**Figure 5:**
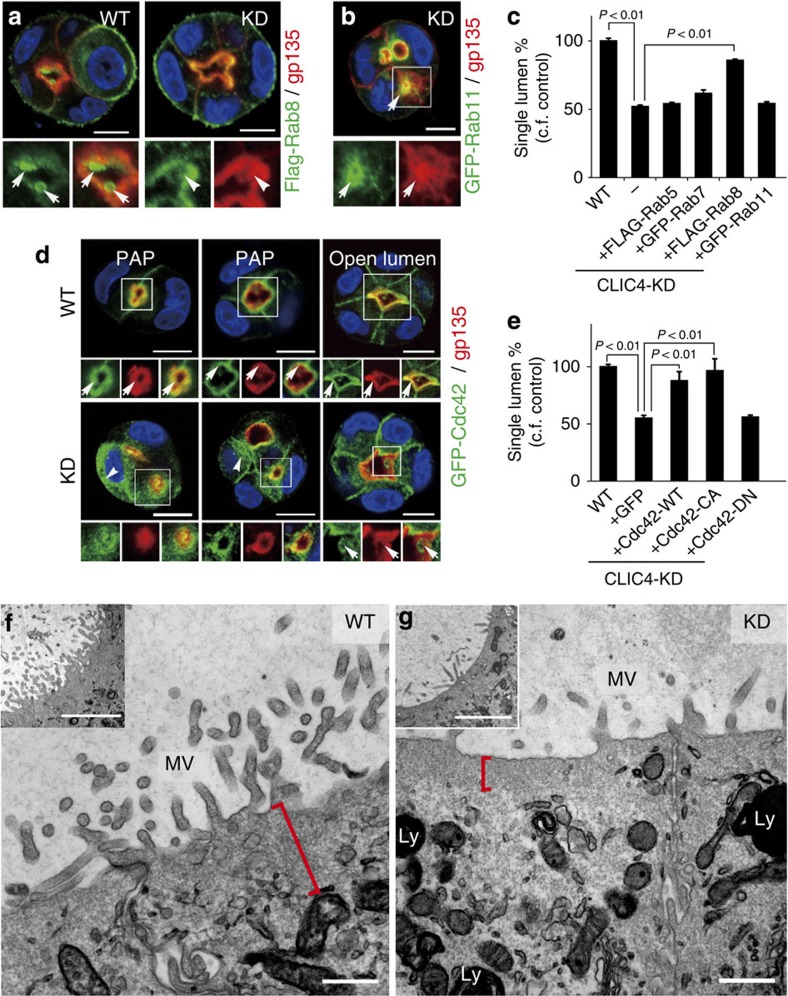
Mechanistic characterization of CLIC4-regulated central lumen formation. (**a**) Examples of the luminal PM location of transfected Flag-Rab8 in WT (arrows) and CLIC4-KD (arrowheads) 3-day cysts. (**b**) A representative CLIC4-KD 3-day cyst transfected with GFP-Rab11a. Arrows point to the overlapping Rab11a and gp135 signals in the ectopic lumen. (**c**) Quantitation of the rescue effect exerted by indicated Rab GTPases on single-lumen formation. The fraction of 3-day cysts containing a single lumen in WT cells was considered as 100%. Error bars represent standard deviation; *P* value by *t*-test; *n*>150; 3 repeats. (**d**) Different stages (1-3-day) of 3D cultures formed by GFP-Cdc42-transfected WT and CLIC4-KD cells. Strong Cdc42 signals were continuously present in gp135-labelled luminal PM in WT cysts (arrows; top panel). GFP-Cdc42 had a diffuse, cytosolic expression pattern in early cysts formed by CLIC4-KD cells (arrowheads, bottom panel); GFP-Cdc42 reappeared on the gp135-enriched luminal PM (arrows, bottom panel). (**e**) Quantification of 3-day cysts formed by CLIC4-KD cells transfected with the indicated plasmids. The fraction of cysts containing a single lumen in WT cells was considered as 100%. Error bars represent standard deviation; *P* value by *t*-test; *n*>170; 3 repeats. (**f**,**g**) Electron micrographs of low- (insets) and high-power views of MDCK WT (**f**) and CLIC4-KD (**g**) cysts cultured for 6 days. The red bracket in (f) marks the subapical cytoplasm of WT cyst containing many endosome-like membrane structures. The red bracket in (**g**) shows that the thin subapical region CLIC4-KD cyst is largely devoid of tubular membrane structures. Ly: electron-dense lysosome. On the surfaces facing both central and ectopic lumens, the mutant cysts developed significantly shorter and sparse MV-like processes. Scale bars, 10 μm (**a**,**b**,**d**); 500 nm (**f**,**g**); 2 μm (inset of **f**,**g**).

We subsequently showed that transfected Flag-Rab8, but not GFP-Rab11a, Flag-Rab5 and GFP-Rab7, was able to target to the luminal PM and restore the central lumen formation of CLIC4-KD cysts (Fig. 5a-c). Furthermore, while transfected GFP-Cdc42 was predominantly on the apical PM in WT cysts at all stages, it was diffuse in the early mutant cysts (Fig. 5d). With time, the membranous GFP-Cdc42 signal reappeared, and this was concomitant with the formation of the central lumen (Fig. 5d). The constitutively active Cdc42(G12V), but not GTPase-deficient Cdc42(T17N), could restore the ability of CLIC4-KD cells to form a central lumen (Fig. 5e). These results collectively suggest that CLIC4 plays a regulatory role in the Rab11a-Rab8-Cdc42-mediated apical exocytosis during luminogenesis.

Next, we determined whether the luminogenic defect seen in CLIC4-KD cells can be overcome with time. At the light microscopic level, the ability of 6-day CLIC4-KD cells to form a single lumen was indiscernible from that of control cells (Supplementary Fig. 5c). However, at the ultrastructural level, we found that the mutant cysts had drastically less and shorter MV compared to the WT (Fig. 5f,g). These electron micrographs also revealed that the subapical cytoplasm of WT cysts, but not CLIC4-KD cysts, was enriched with tubular membrane structures (presumably endosomes) (Fig. 5f,g). These mutants also tend to have more electron-dense lysosomes (Fig. 5g), a phenotype supported by the LysoTracker quantification (Supplementary Fig. 5d).

### CLIC4-associated retromer is important for luminogenesis

The morphological and functional changes of EE, RE and LE resulting from CLIC4 silencing resemble those caused by interfering with the components of retromer-mediated endosomal trafficking^34^,^35^,^36^. Retromer is composed of two subcomplexes, the sorting nexin complex (Snx1/2 and Snx5/6) and the Vps35-Vps29-Vps26 cargo-recognition complex (Supplementary Fig. 6a). On the EE surface, retromer recruits the WASH1 complex and then the Arp2/3 complex to facilitate branched actin formation on microdomains where the selected cargos are concentrated for vesicular delivery^37^,^38^.

**Figure 6:**
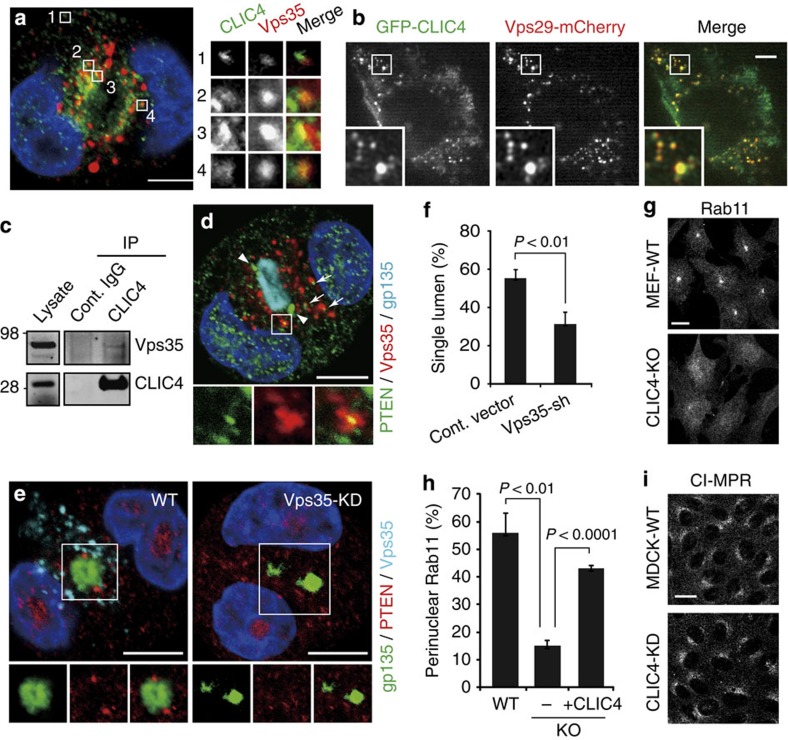
CLIC4 regulates retromer-mediated apical sorting on EE. (**a**) A representative confocal image shows an early (1-day) WT cyst labelled for CLIC4 and Vps35. Enlarged views of the boxed areas (1-4) on the right show the close association between CLIC4 and Vps35 on EE. Pearson's coefficient=0.35±0.04 (*n*=8). (**b**) A representative frame of the time-lapse video taken from a transfected MEF expressing low levels of GFP-CLIC4 and Vps29-mCherry (see Supplementary Movie 1). Enlarged views of the boxed area demonstrate the extensive colocalization of these two molecules. Pearson's coefficient=0.64±0.16 (*n*=11). (**c**) Immunoblots of total membrane lysates of MDCK cells and immunoprecipitates pulled down by using either the host species-matched control (cont.) IgG or anti-CLIC4 antibody. (**d**) A representative image shows an early (1-day) WT cyst triply labelled for PTEN, Vps35 and gp135. Enlarged view of a boxed area highlights the close association between PTEN and Vps35, which was frequently seen in the apical cytoplasm (arrows). Arrowheads show the steady-state PTEN location at TJs. gp135 labelled the nascent luminal PM. Pearson's coefficient (PTEN versus Vps35)=0.28±0.09 (*n*=4). (**e**) Examples of triple-labelled early cysts (1-day) formed by MDCK cells transfected with vector alone or Vps35-shRNA. Enlarged views of the boxed areas are shown. Note the reduction of Vps35 labelling in the Vps35-shRNA transfected cells, as well as the disappearance of PTEN at TJs surrounding the nascent lumen. (**f**) Percentage of 3-day cysts containing a single lumen. Error bars represent standard deviation; *P* value by *t*-test; *n*>450; 3 repeats. (**g**) Immunolabelling of Rab11a. Instead of enriching as the perinulcear punctate in WT MEFs, Rab11a appeared diffuse in the cytoplasm in CLIC4-KO MEFs. (**h**) Percentage of cells (WT MEF, CLIC-KO MEF without (−) or with (+) the transfection of Flag-CLIC4) displaying perinuclear enrichment of Rab11a labelling. Error bars represent standard deviation; *P* value by *t*-test; *n*>250; 3 repeats. (**i**) Similar TGN distribution of CI-MPR was found in WT and CLIC4-KD MDCK cells. Scale bars, 10 μm (**a**,**b**,**d**,**e**,**g**,**i**).

To test the involvement of retromer-mediated trafficking in luminogenesis, we first showed that CLIC4 was present on the Vps35-labelled EE of two-cell stage MDCK cysts (Fig. 6a). Live imaging also showed that GFP-CLIC4 extensively overlapped with the EE-localized Vps29-mCherry (Fig. 6b and Supplementary Movie 1). Finally, co-immunoprecipitation of MDCK membrane-bound lysates (Fig. 6c) demonstrated a physical interaction between CLIC4 and Vps35, although the affinity was not very strong.

Next, we showed that PTEN transiently appeared on Vps35-localized EE of early MDCK cysts (Fig. 6d). Importantly, MDCK cells transfected with Vps35-shRNA (resulting in a perturbed retromer complex^39^) failed to deliver gp135 and PTEN to their respective AMIS/apical PM and TJ locations, and to form a single lumen (Fig. 6e, f and Supplementary Fig. 6b). The requirement of retromer-mediated endocytic trafficking in luminogenesis suggests that further sorting of the cargoes (either internalized from the ECM-contacting surfaces and/or newly synthesized) at the EE is important for the genesis of apical transport vesicles for coalescence.

### CLIC4 regulates EE to RE trafficking

As the WT cyst matured, EEA1/Vps35-labelled EE and Rab11a-labelled RE became spatially resolved in the apical cytoplasm (Supplementary Fig. 6c). By contrast, the physical separation between these organelles was incomplete in the CLIC4-KD cysts; intermediate compartments mixing with these molecules were increasingly observed (Supplementary Fig. 6c). Furthermore, we found that both CLIC4-KD MDCK subconfluent cultures (Supplementary Fig. 6d) and CLIC4-KO mouse embryonic fibroblasts (MEFs) in which CLIC4 was completely depleted (Fig. 6g,h) tended to lose the perinuclear/pericentriolar enrichment of Rab11a, a phenotype that can be rescued by reintroducing Flag-CLIC4 (Fig. 6h). These results collectively indicate that, when CLIC4 is suppressed, the delivery of Rab11a from the EE tubule to the RE is compromised, so that Rab11a failed to mediate the process to form RE.

On the other hand, unlike the dispersed pattern in the VPS35-KD cells^40^,^41^, the cation-independent mannose 6-phosphate receptor (CI-MPR) had a relatively normal TGN location in the CLIC4-KD cells (Fig. 6i). These results suggest that CLIC4 participates in EE-to-RE delivery of Rab11a, but not in EE-to-TGN retrograde transport of CI-MPR.

### CLIC4 regulates actin network formation on EE

**Figure 7:**
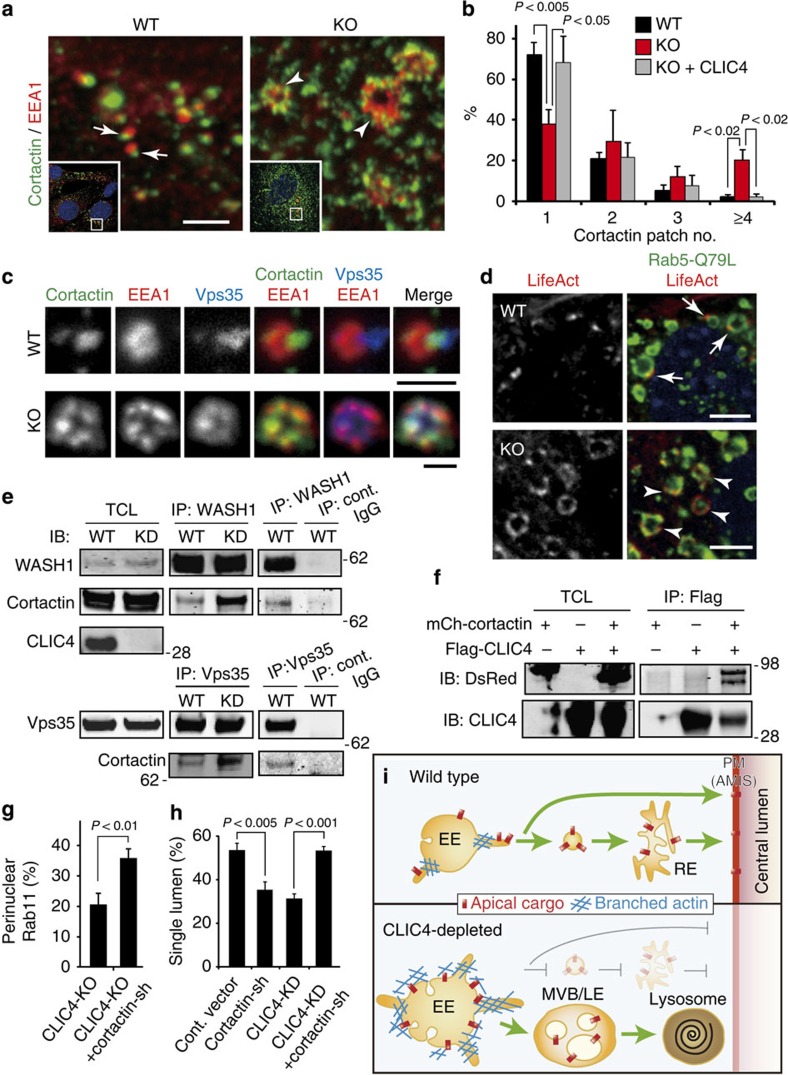
CLIC4-cortactin association modulates actin assembly on EE surface. (**a**) Low- (insets) and high-power views of MEFs stained for cortactin and EEA1. Arrows point to EEs displaying one cortacin-labelled ‘patch' in WT. Arrowheads point to enlarged EEs decorated with multiple cortactin-labelled ‘patches' in KO. (**b**) Fractions of MEFs (WT, CLIC4-KO without or with transfected Flag-CLIC4) decorated with 1-4 (or more) cortactin-positive patches on EE. Error bars represent standard deviation; *P* value by *t*-test; *n*>145; 3 repeats. (**c**) Representative images of triply labelled EE of WT and CLIC4-KO MEF. (**d**) MEFs expressing low levels of transfected Flag-Rab5Q79L and mCherry-LifeAct. Arrows and arrowheads point to the actin patches on the EE of WT and KO cells, respectively. (**e**) Total cell lysates (TCL) from the CLIC4-shRNA inducible MDCK cells treated with (KD) or without (WT) doxycycline were subjected to immunoprecipitation using the anti-WASH1 or anti-Vps35 antibody. Host species-matched IgGs were used for negative controls (cont.). (**f**) Immunoprecipitation of HEK cells transfected with mCherry-cortactin and/or Flag-CLIC4 using the anti-Flag antibody, and subsequent immunoblottings using anti-DsRed (crossreacts with mCherry) and anti-CLIC4 antibodies. (**g**) Quantification of CLIC4-KO MEF (without or with cortactin-shRNA transfection) that expressed perinuclear enrichment of Rab11a. Error bars represent standard deviation; *P* value by *t*-test; *n*>100; 3 repeats. (**h**) Quantification of the single-lumen formation in (3-day) MDCK cysts transfected with various shRNA. The cortactin-shRNA (or control vector) was transfected into either the WT or the CLIC4-KD MDCK line described in (**e**). Error bars represent standard deviation; *P* value by *t*-test; *n*>550; 3 repeats. (**i**) Working model. CLIC4 regulates apical trafficking by modulating the EE's surface branched actin, required for organelle microdomain remodeling, cargo molecule sorting and/or tubular fission. Endosomal vesicles harbouring apical cargo may directly fuse onto, or transit through RE *en route* to apical PM (AMIS). CLIC4 depletion abnormally increases branched actin on EE surface, and interferes with membrane remodelling, cargo sorting and apical vesicular delivery. These abnormalities together may enhance the membrane's involution, rendering more LE formation; apical elements crucial for RE genesis tend to be shunted to the LE/lysosomal degradation pathway. Scale bars, 2 μm (**a**), 1 μm (**c**), 5 μm (**d**).

Similar to Vps35-KD and WASH1-KO cells^35^,^42^, both CLIC4-KD MDCK cells and CLIC4-KO MEFs (Fig. 7a) had structurally abnormal EE with an enlarged, collapsed and/or bubbled appearance. However, immunostaining results showed that CLIC4 was not critical for the recruitment of the retromer complex (Vps35, Snx2; Supplementary Fig. 7a) and the WASH1 complex (Supplementary Fig. 7b) onto the EE surface.

Previous studies showed that the dynamic actin and branched actin networks on EE surfaces are important for membrane remodelling, which is crucial for selective cargo sorting and EE shape control^16^,^17^,^36^,^37^,^43^. We found that the majority of WT EE was decorated with 1-2 globular-shape ‘patches' expressing cortactin, a marker for the branched actin network^37^,^44^. In the mutant cells the number of cortactin ‘patches' on EE (which had irregular tubular and/or tuft shape) was drastically increased (Fig. 7a,b). Furthermore, on the EE of WT cells, cortactin was typically confined to the EEA1-devoid microdomains and proximal to the Vps35-labelled tubules, whereas it became broadly distributed on EE surfaces and extensively overlapped with EEA1 and Vps35 in CLIC4 mutant cells (Fig. 7c).

By ectopically expressing Rab5Q79L, we found that those atypical cortactin (Supplementary Fig. 7c) or actin (indicated by the transfected mCherry-LifeAct; Fig. 7d) patterns were specifically detected in the enlarged EE of CLIC4-KO MEFs, but not in WT MEFs. Thus, the elevated actin assembly caused by CLIC4 depletion could not be explained simply by the increased size of this organelle.

To quantify the changes of the level of the EE-associated cortactin, we showed that, while the total cortactin level was unaffected, the amount of cortactin specifically co-immunoprecipitated with Vps35 or WASH1 was increased (∼3-fold; Fig. 7e). To further probe a possible CLIC4-cortactin interaction, we showed that GFP-CLIC4 and mCherry-cortactin were extensively overlapped in transfected MEF cells (Supplementary Fig. 7d). Furthermore, Flag-CLIC4 was able to co-immunoprecipitate mCherry-cortactin from the HEK cell lysates (Fig. 7f). Mapping studies using various cortactin truncates showed that the region comprising aa 82-330 (actin-binding domain) was sufficient to bind CLIC4 (Supplementary Fig. 8a). Subsequently, we showed that purified recombinant cortactin (aa 82-330) fragments were able to specifically pull down purified CLIC4 (Supplementary Fig. 8b-d). The converse experiments showed that purified glutathione transfer protein (GST) fusion of CLIC4, but not GST alone, was also able to pull down the purified cortactin fragment (Supplementary Fig. 8e). These results suggest that the interaction between CLIC4 and cortactin is direct.

Finally, we addressed the question of whether the excess cortactin on EE contributes to the phenotypes seen in the CLIC4-mutant cells. To this end, we showed that the perinuclear enrichment of Rab11a in CLIC4-KO MEFs could be significantly restored by the transfection of validated cortactin-shRNA (Fig. 7g and Supplementary Fig. 8f). Furthermore, like CLIC4-KD cells, cortactin-KD MDCK cells had impaired ability to form single-lumen cysts (Fig. 7h). Importantly, cortactin-shRNA transfection was able to overcome the defect in central lumen formation in CLIC4-KD cysts (Fig. 7h and Supplementary Fig. 8g). These results collectively suggest that CLIC4 regulates luminogenesis by modulating the retromer- and branched actin-mediated EE vesicular trafficking.

## Discussion

Consistent with its role in apical exocytosis, CLIC4 is associated with the apical transport cargoes, the EE, the RE, and the apical surface during early luminogenesis of MDCK cyst cultures. Our data suggest that Rab11a and PTEN are the cognate cargoes whose apical delivery is regulated by CLIC4. Silencing CLIC4 inhibits central lumen formation by perturbing localization of Rab11a to the apical RE and PTEN to the nascent lumen PM without affecting the basolateral protein targeting. Mistargeting of PTEN could lower the local enrichment of PI(4,5)P2, and, hence, the initial development of AMIS for future lumen growth. On the other hand, impaired vesicular transport of Rab11a (and its associated molecules) from EE could impede the biogenesis of RE. Since Rab11a is important for protein sorting (at the apical RE) and vesicle docking (at the apical PM)^3^,^45^,^46^, reduced Rab11a on the residual REs likely further compromises the apical surface delivery. CLIC4 is likely to play a similar regulatory role in apical exocytosis of PT epithelial cells. Studies carried out both *in vivo* and *in vitro* imply that megalin is a cognate cargo. Further supporting this notion, we showed that the apical surface targeting of transiently transfected p75 neurotrophin receptor in LLC-PK1 cells^47^ was also disrupted when CLIC4 level was suppressed (Supplementary Fig. 9a). It is conceivable that increased LE/lysosome is causally related to the defective RE/surface transport from EE (Fig. 7i).

Consistent with our proposal that CLIC4 modulates retromer-mediated EE membrane trafficking, CLIC4-mutant and Vps35-depleted cells share several common phenotypes. These include the structural change of EE/LE/lysosomes^34^,^35^,^36^,^42^, defective apical delivery (of PTEN, gp135, Rab11a) and luminogenesis. This and our other findings collectively suggest that CLIC4 is a novel regulator for selective retromer-mediated endocytic trafficking toward the apical route without impacting the endosome-to-TGN retrograde transport. While actin dynamics on the EE surface is known to be important for selective cargo transport^36^,^38^,^43^, understanding of its regulation, especially the negative regulatory elements, is rather limited. Here we show that CLIC4 depletion enhanced branched actin expression on EE. Since cortactin promotes and stabilizes the branched actin filament network^48^,^49^, abnormally high level of cortactin on the EE of CLIC4-depleted cells could lead to a change in the pattern of branched actin that, in turn, interferes with its ability to form (and/or maintain) microdomains for cargo sorting, and/or severing the sorting tubules. This model was supported by our functional evidence that cortactin suppression is able to rescue CLIC4-KD-mediated phenotypes.

Probing further the dynamics and regulation between CLIC4, actin and cortactin, we found that, consistent with previous studies^15^, CLIC4 itself did not bind to F-actin in co-sedimentation assay (Supplementary Fig. 9b). Furthermore, CLIC4 did not detectably affect the amount of cortactin co-pelleted with F-actin (Supplementary Fig. 9c,d), indicating that CLIC4 is not directly involved in loading of cortactin onto the F-actin filament. Thus, it remains to be investigated whether CLIC4 modulates some other aspect(s) of cortactin in actin organization (e.g., crosslinking, nucleation)^50^, and/or its interaction with endosomal lipid membranes^51^,^52^. Note that CLIC4 has also been shown to directly bind to 14-3-3 and dynamin^15^, both molecules known to regulate actin dynamics as well^50^,^53^,^54^. Furthermore, dynamin binds to the C-terminus of cortactin. Therefore, CLIC4 interacts with cortactin and the actin network in a multivalent fashion; CLIC4 silencing resulting in altered cortactin expression on endosomes could also be affected by molecules that bind both CLIC4 and cortactin. Since the formation of branched actin networks requires a highly orchestrated balance of nucleation, branching and turnover, the precise role (likely multifactorial) of CLIC4 in maintaining just the right level of branched actin requires further investigation.

The close functional relationship between CLIC4 and cortactin appears to be evolutionarily conserved. In *C. elegans* both *exc-4* (the single *clic* homologue) and *exc-5* mutants cause similar excretory cysts. The *exc-5* gene encodes a mammalian homologue of Facial–Genital Dysplasia 1, a guanine nucleotide exchange factor of Cdc42 that is known to bind to cortactin, and stimulates Arp2/3-mediated actin polymerization *in vitro*^55^. The importance of Cdc42 in regulating cortactin/actin dynamics may contribute to its ability to rescue CLIC4-KD-mediated luminogenesis. On the other hand, small Rho GTPases are important for activating several actin nuclear promoting factors, including the WASP/WAVE/WASH1 family proteins^16^,^56^. CLIC4 is neither a guanine exchange factor nor a guanine-activating protein of Rab GTPase; thus, it is unlikely that it regulates their GTPase activity. Yet, the membrane-binding ability of CLIC4 can be enhanced by RhoA activation^12^. Future characterization of the interaction between CLIC4, Rho GPTase(s) and its other binding partners may delineate the apical cue(s) and/or the mechanism controlling the directionality of the CLIC4-regulated transport.

The coincidence that both CLIC4 *(exc-4 homologue)* and Facial–Genital Dysplasia 1 (*exc-5 homologue)* are functionally related to cortactin accentuates the importance of spatial-temporal regulation for branched actin formation during epithelial tubulogenesis. While previous studies in lower organisms have linked the structural (i.e., apical membrane skeleton) role of actin to luminal morphogenesis^57^, here we emphasize that actin on the endosomes has a vital role in apical vesicular trafficking during *de novo* lumen formation.

The PT nephrons in adult CLIC-KO kidneys undergo dilation, resembling the key feature of Fanconi syndrome^58^,^59^. Two familial forms of Fanconi syndrome, Dent's disease and Lowe syndrome, have been linked to mutations of the CLCN5 chloride channel/exchanger^60^ and inositol polyphosphate 5-phosphatase^61^, respectively. Coincidently, both of these molecules are involved in the endolysosomal vesicular trafficking of PT cells^62^,^63^,^64^,^65^. Furthermore, the expression level of CLIC4 has been recently linked with the susceptibility of acute kidney injury^27^; PT dilation and loss-of-MV are hallmarks of early-phase acute kidney injury^66^,^67^. Finally, our observation that CLIC4 is important for both initial development of proper lumen (i.e., cavity, membrane specialization) and the lumen size/shape control of the epithelial tubes indicates that these two cellular processes share common mechanisms, such as actin-mediated endolysosomal trafficking and membrane remodelling.

We further speculate that the defective MV morphogenesis is relevant to the CLIC4-KO PT dilation (Supplementary Note 2). CLIC4's role in the MV specialization is conserved in both retinal pigment epithelial^68^ and renal PT cells. Coincidentally, the intestinal epithelial cells of Rab8-KO mice also exhibit malformed MV (and excess lysosomal degradation)^69^. We are tempted to propose that CLIC4- and Rab8-regulated apical exocytosis is functionally involved in MV morphogenesis.

It remains perplexing why a cytosolic protein CLIC4, when heterologously expressed, can generate a weak (low selective) anion conductance on cell surfaces^70^. Our finding that CLIC4's expression level correlates with its exocytic activity raises the tantalizing possibility that increased CLIC4 expression promotes the PM insertion of other bona fide (chloride) channel(s).

There has been a fast-growing interest in the CLIC family of proteins. The divergent evolution of the CLIC family proteins (i.e., one for nematode, two for fly and six for mammals) may reflect the need for their different functions in higher organisms. Emerging evidence has linked CLIC3 to the surface recycling of the integrin receptor from LE/lysosome in ovarian cancer cells^71^,^72^, and CLIC1 to the phagosomal acidification in macrophage^73^. Taking together with CLIC4's role in EE to RE and to apical PM, we propose that CLIC proteins represent a novel family of proteins involved in vesicular trafficking on distinct membrane compartments.

## Methods

Reagents including plasmids, antibodies (concentrations and dilutions used) and cell lines used in this study are detailed in the Supplementary Methods.

### Renal histology and ultrastructural analyses

Age-matched homozygous CLIC4 KO mice and their WT littermates were compared. In some experiments, *clic4*^*f/f*^ mice were used as ‘WT' in this paper as they did not exhibit any detectable renal phenotype compared to C57BL/6J mice. For light microscopic analyses, kidneys were fixed in 10% neutral buffered formalin followed by paraffin embedding, sectioning and standard methods for PAS staining. For immunostaining, deparaffinized renal sections were subjected to antigen retrieval Tris/EDTA pH 9.0 buffer, at 97 °C for 30 min prior to the antibody incubation. Biotinylated LTA (Vector Labs) was visualized by Alexa-dye-conjugated streptavidin. The WT and mutant renal sections were stained and imaged on a Leica TCS SP2 spectral confocal system in parallel under the same parameter setting. At least six mice were examined for each staining condition.

For ultrastructural analysis, metanephros were cut by vibrotome, permeabilized by the ‘freeze-thaw' method, incubated with anti-CLIC4 antibody and post-fixed with 2% glutaraldehyde for 10 min after the secondary antibody incubation. The immunolabelled sections were then processed for silver enhancement, OsO_4_ fixation, and dehydration. The sections were then flat embedded in Epon, cut into 70-nm-thick ultrathin sections, counterstained and examined on a Philips CM10 electron microscope. Conventional EM was carried out using the same procedures except that the immunolabelling steps were omitted.

### MDCK 3D cultures and transmission EM

Three thousand cells were singly resuspended in 200 μl of DMEM medium with 5% fetal bovine serum and 2% Matrigel (Invitrogen), seeded onto the eight-well Lab-Tek chamber (NUNC) pre-coated with 100% Matrigel and incubated for the indicated lengths of time. For cortactin or Vps35 KD, the corresponding shRNA was transiently transfected into MDCK cells and cultured for 1 day (for cortactin-shRNA) or 3 days (for Vps35-shRNA) prior to Matrigel plating.

For EM analysis, 6-day cyst cultures grown on Transwell filters (Corning; 6.5 mm diameter, polyester 0.4 μm pore size) were fixed with 3.75% acrolein plus 4% paraformaldehyde in 0.1 M cacodylate buffer (pH 7.4) at room temperature for 10 min, and then postfixed with 2.5% glutaraldehyde plus 4% paraformaldehyde in 0.1 M sodium cacodylate buffer (pH 7.4) for 2 days at 4 °C. The samples were then *en block* counter-stained, embedded into Embed-812, cut and examined on a Philips CM10 electron microscope.

### Immunostaining

Immunostaining was carried out using a standard protocol. Briefly, cells were washed twice with PBS-C/M (PBS containing 0.2 mM CaCl_2_ and 2 mM MgCl_2_), fixed with 4% paraformaldehyde for 10 min, washed and quenched with 50 mM NH_4_Cl for 10 min. After 1 h incubation with the blocking buffer (PBS-C/M with 0.5% BSA, 0.2 mg ml^−1^ Na-azide, 0.3 μM DAPI and 0.25% Triton X-100), cells were incubated with primary antibodies in the blocking solution at room temperature for 1 h. Cells were then washed three times with PBS-C/M, followed by 1 h incubation with secondary antibodies in the blocking solution. Samples were mounted with ProLong Gold Antifade reagent (Life Technologies). For MDCK 3D cultures, the incubation times were extended to get better reagent penetration: fixation for 20 min, quench for 15 min, primary antibodies for overnight and secondary antibodies for 2 h.

For F-actin staining, Alexa-conjugated Phalloidin (Molecular Probes) was added during secondary antibody incubation. For cortactin staining, pre-extraction was performed prior to the standard staining procedure. Briefly, cells were rinsed with PBS-C/M and incubated with pre-extraction buffer (80 mM PIPES, pH 6.8, 1 mM EGTA, 3 mM MgCl_2_, 30% glycerol, 0.02% saponin, 2 μM phalloidin) for 1 min at room temperature, then washed twice with the wash buffer (80 mM PIPES, pH 6.8, 1 mM EGTA, 3 mM MgCl_2_, 30% glycerol) and fixed for 10 min with 4% paraformaldehyde in wash buffer.

### Microscope and quantification analysis

Fluorescent images were acquired with a × 63 objective on a Leica TCS SP2 spectral confocal system. All images shown were from ∼0.24-μm-thick sections for subconfluent cells and from ∼0.3 to 0.4-μm-thick sections for MDCK 3D cultures unless otherwise indicated. For live movies, the transfected MEFs were plated onto glass-bottom dishes (Greiner Bio-One) and cultured with DMEM containing 10% FBS overnight. Before recording, cells were briefly washed with PBS and incubated with pre-warmed recording buffer (Hank's balanced salt solution supplemented with 1% FBS and 4.5 g l^−1^ glucose). Movement of GFP-CLIC4 and Vps29-mCherry-containing vesicles was captured by a wide-field fluorescence microscope (Axio Observer.Z1; Zeiss) equipped with the Plan-Apochromat × 63/1.4 oil immersion objective, the AxioCam HRm camera, the CO_2_ Module S and the TempModule S (Zeiss). For presentation purposes, images were processed by Photoshop (CS3 or newer version, Adobe Systems Inc.) or Image J (1.47v). Some images were edited with a deconvolution software (Deconvolution Lab) plugged in Image J using Tikhonov-Miller algorithm. The level of colocalization of the two proteins (Pearson's correlation) was analysed by using the Coloc2 software plugged in Image J. The linear scanning profile of LTA signals was also depicted with the program built in Image J.

For the measurement of cortical thickness of mouse metanephors, light micrographs of the E15 renal sections were imaged under a × 4 lens; the cortical length of one section was measured concentrically from multiple locations and then averaged to obtain the mean. The overall mean values were further averaged from multiple sections among six animals in each group.

Solid cortactin patches and tubules overlapped with EEA1-positive endosome were counted from the images obtained above. The Rab11a distribution was examined under Axioskop2 (Zeiss), and cells with a typical pole punctate and/or arc-shaped perinuclear enrichment of Rab11a signals were considered as positive. To quantify lumen formation, more than 30 random fields of a 2-3-day 3D culture were monitored under a × 40 lens and counted by eye. Criteria for normal lumens were judged by a clear gp135/phalloidin labelling at the interior surface. Signals weak, absent or in small multi-spaces are considered abnormal lumens. Error bars represent standard deviation of three independent experiments, and statistics were conducted by two-tailed Student's *t*-test.

### Membrane fractionation and immunoprecipitation

Pelleted MDCK cells from a 15-cm confluent dish culture were resuspended in 4 ml of homogenization buffer (10 mM Tris-Cl, pH 7.4, 0.25 M sucrose, 2 mM MgCl_2_, 1 mM EDTA, protease inhibitor cocktails and phosphatase inhibitors) and homogenized by several passages through a ball-bearing homogenizer. The post-nuclear supernatants after 800*g* spinning (10 min at 4 °C) were further centrifuged at 220,000*g* (Beckman TLA 100.3 rotor) for 40 min at 4 °C. The membrane-rich pellet fraction was washed with PBS once, and solubilized in 600 μl of the buffer (PBS containing 1% Triton X-100, 40 mM *N*-ethylmaleimide, protease inhibitor cocktails and phosphatase inhibitors) for 30 min at 4 °C with gentle rotation. The resulting solution was centrifuged at 90,000*g* for 10 min at 4 °C, and the supernatant obtained from this step was collected as the membrane lysate.

The membrane lysate and, in some experiments, the total cell lysates in RIPA buffer (50 mM Tris pH 7.4, 150 mM NaCl, 0.5% NP-40, 0.5% Na-deoxycholate, 0.1% SDS, 1 mM EGTA, proteinase inhibitor cocktails, phosphatase inhibitors, 1 mM PMSF, 5% glycerol) were subjected to immunoprecipitation using Dynabeads Protein G (Invitrogen) performed as described in the manufacturer's protocol. After incubation overnight at 4 °C, the unbound proteins were washed twice using the same lysis buffer, followed by two further washes with PBS containing 0.01% Tween-20. The bound fractions were then cooked and separated by 4-12% SDS-PAGE and transferred to nitrocellulose membranes (Life Technologies). Following incubation with appropriate primary and IRDye-conjugated secondary antibodies, proteins were detected with the Odyssey infrared-fluorescence imager (LI-COR). For the cortactin-CLIC4 interaction assay, transiently transfected HEK 293T cells were harvested and then subjected to the immunoprecipitation assay as described above. All of the original scan images of gels and immunoblots are shown in Supplementary Fig. 10.

## Additional information

**How to cite this article:** Chou, S.-Y. *et al.* CLIC4 regulates apical exocytosis and renal tube luminogenesis through retromer- and actin-mediated endocytic trafficking. *Nat. Commun.* 7:10412 doi: 10.1038/ncomms10412 (2016).

## Supplementary Material

Supplementary Information Supplementary Figures 1-10, Supplementary Notes 1-2, Supplementary Methods and Supplementary References.

Supplementary Movie 1 Close association between GFP-CLIC4 and Vps29-mCherry on endosomes. Time-lapse video images taken every 2 seconds for 2 minutes at 37°C from a MEF cell transfected with GFP-CLIC4 and Vps29-mCherry. Note that GFP-CLIC4 and Vps29-mCherry not only colocalized, but also moved together on the same membrane structures. Still images from a representative time frame are shown in Fig. 6b. Scale bar=10 μm.
