# Supplementary material for: CLIC4 regulates apical exocytosis and renal tube luminogenesis through retromer- and actin-mediated endocytic trafficking
Source: Nat Commun. 2016 Jan 20;7:10412. doi: 10.1038/ncomms10412 (PMC4736046; doi:10.1038/ncomms10412)
Supplement: Supplementary Information — Supplementary Figures 1-10, Supplementary Notes 1-2, Supplementary Methods and Supplementary References. [file ncomms10412-s1.pdf]

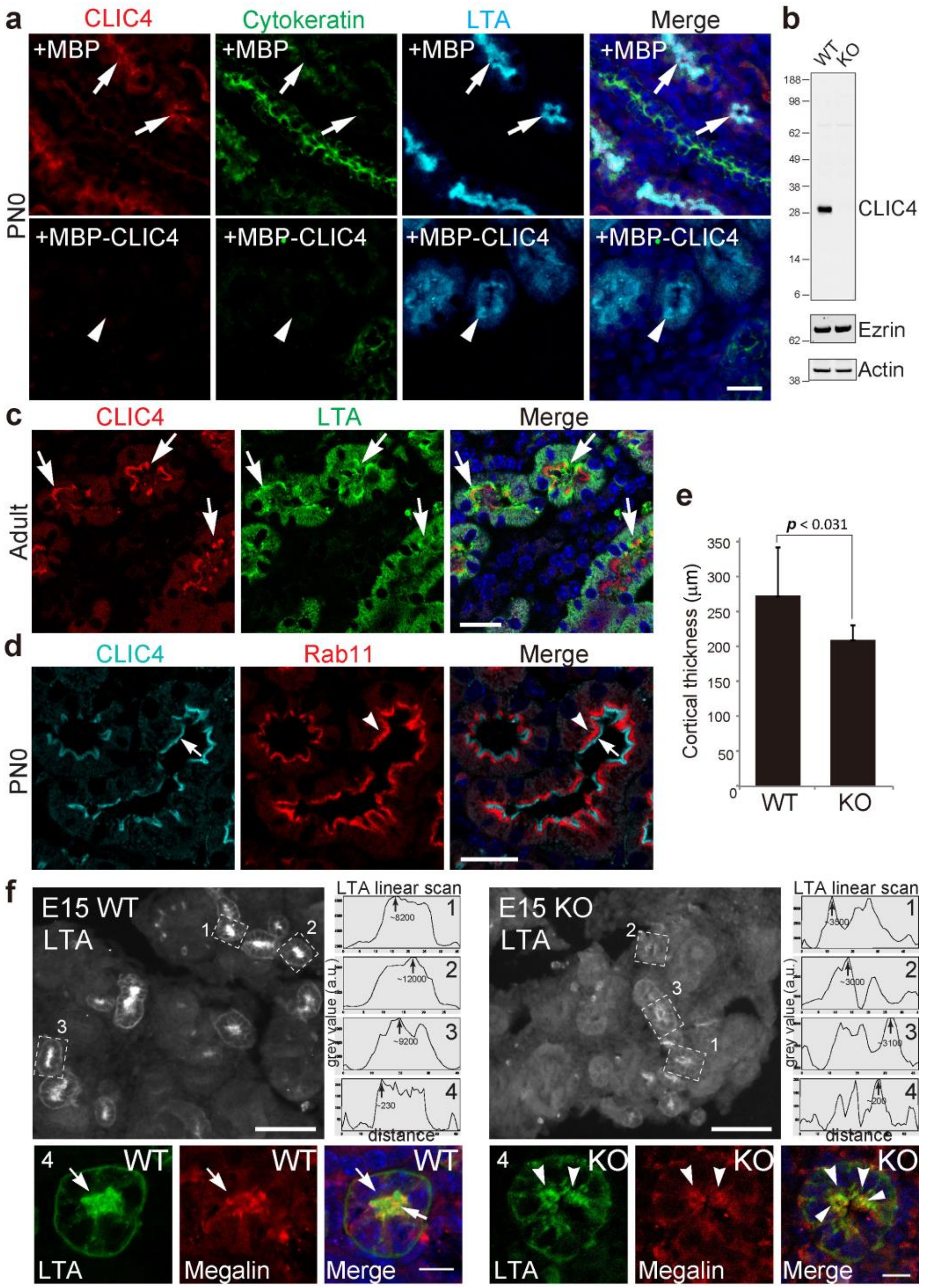

Supplementary Figure 1 CLIC4 expression and CLIC4-KO metanephros abnormality

(**a**) Demonstration of CLIC4 antibody immunospecificity. CLIC4 rabbit antibody were first preabsorbed with either maltose binding protein (MBP) or MBP-CLIC4 fusion protein[1]. The antibody mixtures were then incubated with PN0 mouse renal paraffin sections costained with cytokeratin and LTA. At this developmental stage, CLIC4 was particularly enriched in the LTA-positive, cytokeratin-negative PTs of WT mice (arrows); the PT labeling of CLIC4 was specifically lost when the anti-CLIC4 antibody was mixed with MBP-CLIC4 (arrowheads).

(**b**) Immunoblots of renal lysates harvested from WT and CLIC4-KO PN0 mice.
(**c**) CLIC4 and LTA labeling of PN 3-weeks-old WT mouse renal cortex. Arrows point to CLIC4 enriched at the apical MV brush borders of LTA-labeled PTs. Note that the adult PTs had cytoplasmic green autofluorescence [2].
(**d**) CLIC4 and Rab11a immunolabeling of PN0 PTs. Arrows and arrowheads point to CLIC4's and Rab11a's luminal/subluminal signals, respectively.
(**e**) Cortical thickness of E15 WT and CLIC4-KO metanephros. *p* value by *t* test, n = 10 sections for WT (6 animals); n = 11 sections for CLIC4-KO (6 animals). Error bars represent standard deviation.
(**f**) Top left panels: Low-power views depicting the LTA staining of E15 metanephros from WT (left) and CLIC4-KO (right) mice. Bottom panels show high-power confocal images of a representative pre-PT in WT and KO labeled for megalin and LTA. Arrows point to the co-enrichment of megalin and LTA in the center lumen of WT. Arrowheads point to the displaced LTA and megalin signals in mutant. Top right panels: Linear scanning profiles of LTA labeling of four boxed areas from the left and bottom panels. Maximum value in arbitrary unit (a.u.) of each line profile was pointed out by an arrow. Note that while the WT pre-PTs have LTA signal concentrated in central lumens, the LTA signals are more scattered in KO pre-PTs.
Scale bars = 20 μm (a, c, d); 100 μm (upper panels of f); 10 μm (lower panels of f).

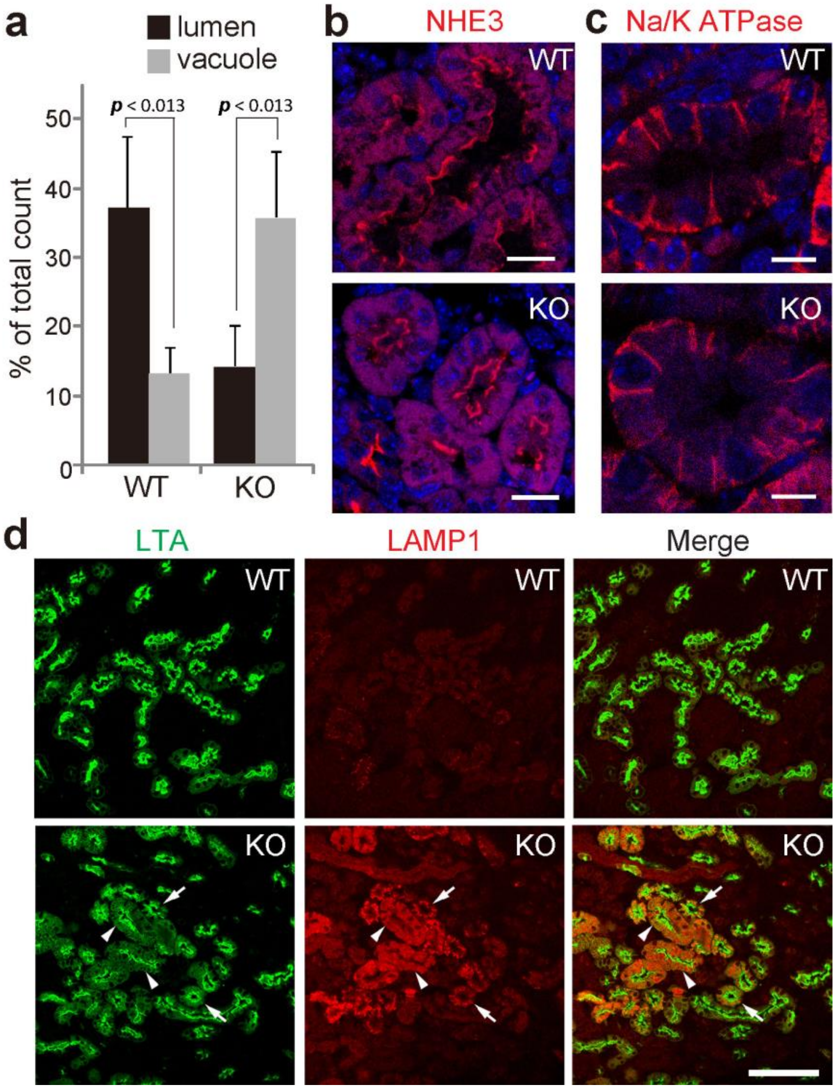

Supplementary Figure 2 The phenotypic characterization of developing PTs in CLIC4-KO mice

(**a**) The histogram shows the percentage of renal cortical tubules in PN0 (WT and CLIC4-KO) mice that had intense PAS labeling at the luminal PM vs. at intracellular vacuoles. Error bars stand for standard deviation. *p* value by *t* test, n = 4 animals for both WT and CLIC4-KO mice.

(**b**) Example of immunolabeling of NHE3 in the PN0 PTs of WT and CLIC4-KO mice.

(**c**) Example of immunolabeling of Na/K-ATPase in PN0 PTs of WT and CLIC4-KO mice.

(**d**) The cortical renal section of PNO WT and KO mice was labeled with LTA and LAMP1. Both the number of PT expressing LAMP1 and the intensity of LAMP1 labeling were greatly increased in the CLIC4-KO mice compared to the WT. Some of the LAMP1 signals had a granular appearance (arrows), whereas others appeared to be diffused throughout the cytosol (arrowheads).

Scale bars = 20 μm (b); 10 μm (c); 100 μm (d).

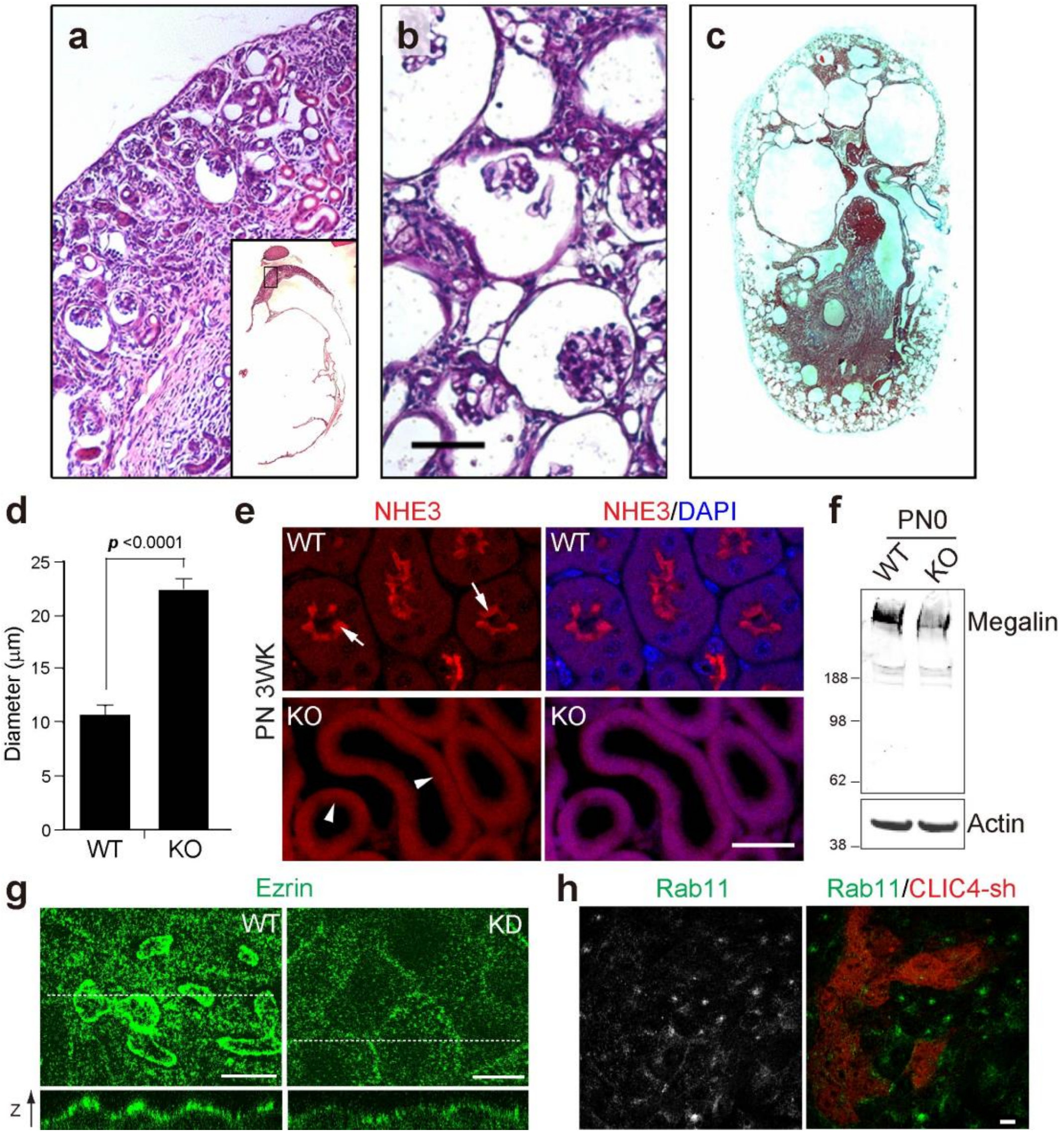

a
b
c
d
p <0.0001
Diameter (μm)
25
20
15
10
5
0
WT
KO
e
NHE3
NHE3/DAPI
WT
WT
PN 3WK
KO
KO
f
PN0
WT
KO
Megalin
188
98
62
Actin
38
g
Ezrin
WT
KD
Z
h
Rab11
Rab11/CLIC4-sh

Supplementary Figure 3 Postnatal CLIC4-KO kidneys exhibit various histological pathologies
(**a**) Hematoxylin and eosin staining shows a mutant kidney displaying severe hydronephrosis (inset); the remaining renal cortex was poorly developed with few recognizable tubules.
(**b**) Hematoxylin and eosin staining of a mutant kidney displaying severe glomerular cysts.
(**c**) Trichrome staining of a mutant kidney displaying severe cortical cysts, a phenotype that resembles the polycystic kidney.
(**d**) Measurement of the diameter of the PT lumens in WT and CLIC4-KO mice. The narrowest region between two cell surfaces (at the base of MV in the case of WT) of an open lumen is taken as the diameter. n = 100 among 3 mice for each group, *t*-test; Error bars represent standard deviation.
(**e**) Representative confocal images of 3-week-old cortical sections of WT and CLIC4-KO kidneys labeled for NHE3. The MV NHE3 labeling in the WT (arrows) was largely absent in the mutant (arrowheads).
(**f**) Immunoblots of WT and CLIC4-KO PN0 renal lysates probed with anti-megalin and actin antibodies.
(**g**) LLC-PK1(CL4) cells stably expressing doxycycline-induced CLIC4-shRNA was treated without (WT) or with (KD) doxycycline and stained for ezrin. Representative confocal images captured under the same conditions were shown. Upper panels were x-y views, and the lower panels were x-z scans across the dotted lines shown in x-y views. An arrow points to the apical side.
(**h**) LLC-PK1(CL4) cells transiently expressing CLIC4-shRNA/RFP were immunolabeled for Rab11a (green). Noted that untransfected, but not the “red” transfected, cells had prominent perinuclear Rab11a labeling.
Scale bars = 100 μm (b); 25 μm (e); 10 μm (g, h).

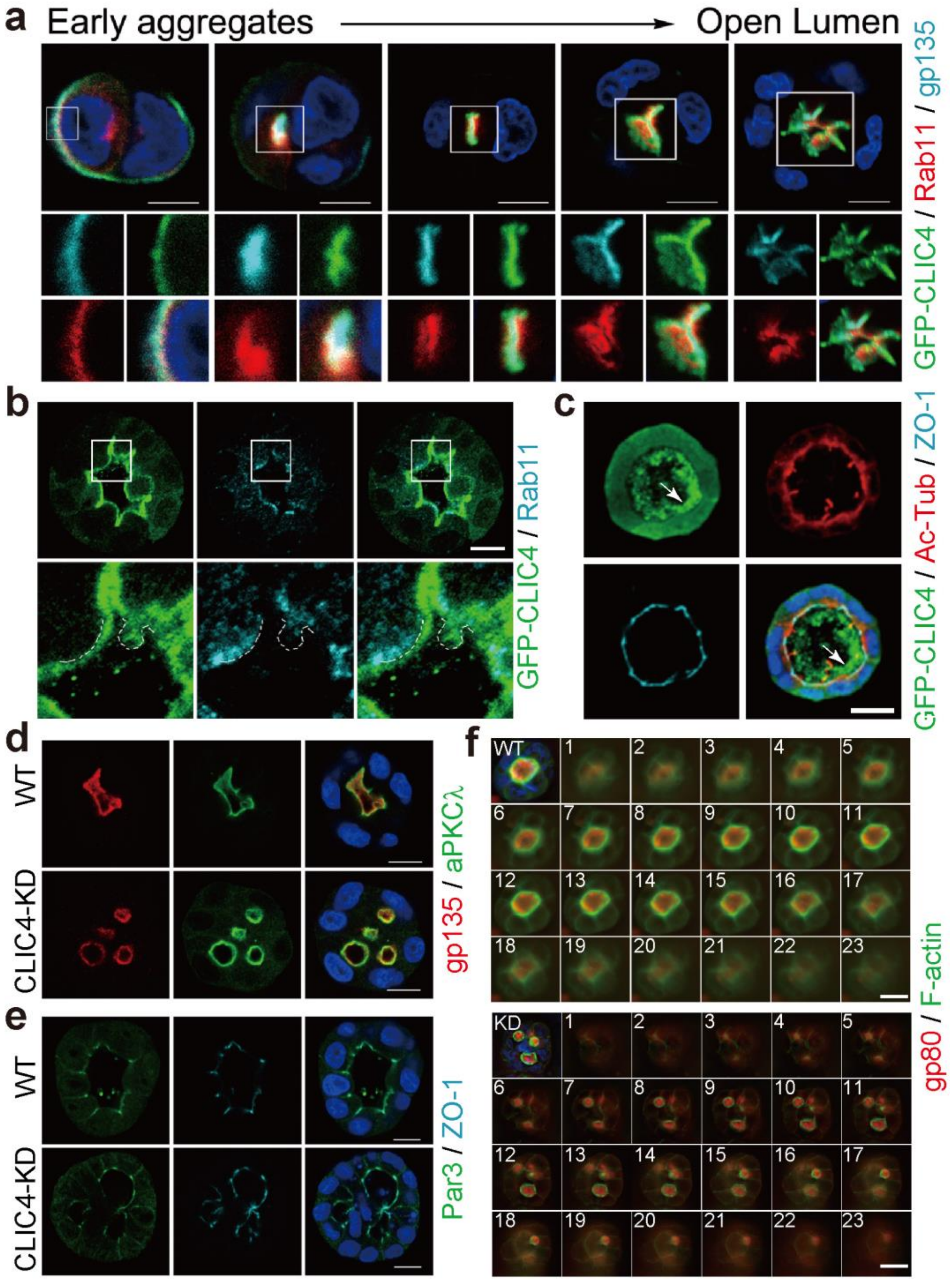

Supplementary Figure 4 Expression of GFP-CLIC4 and localization study of CLIC4-KD cysts
(**a, b**) Different stages (12h ~3 days) of 3D cultures formed by the MDCK cells stably expressing GFP-CLIC4 that were immunolabeled for Rab11a and gp135. Magnified views of boxed areas are also shown. Dotted line in (b) demarcates the border between the luminal PM and subapical cytoplasm.
(**c**) The luminal membrane in a 6-day cyst is outlined by the labeling of acetylated α-tubulin (Ac-Tub) and ZO-1; an arrow points to the GFP-CLIC4 expressed on the MV facing the lumen.
(**d, e**) Confocal images of 3-day cyst cultures formed by WT or CLIC4-KD cells co-labeled for the indicated markers.

(**f**) Serial stack images of a representative 3-day cyst formed by the WT and CLIC4-KD cells stained for gp80 and F-actin (using Alexa-488-Phalloidin). The overall projected images are shown at the top-left corners, followed by serial Z-sections from Z1 to Z23 with 1 μm increment.
Scale bars = 10 μm (a, b, d, e); 20 μm (c, f).

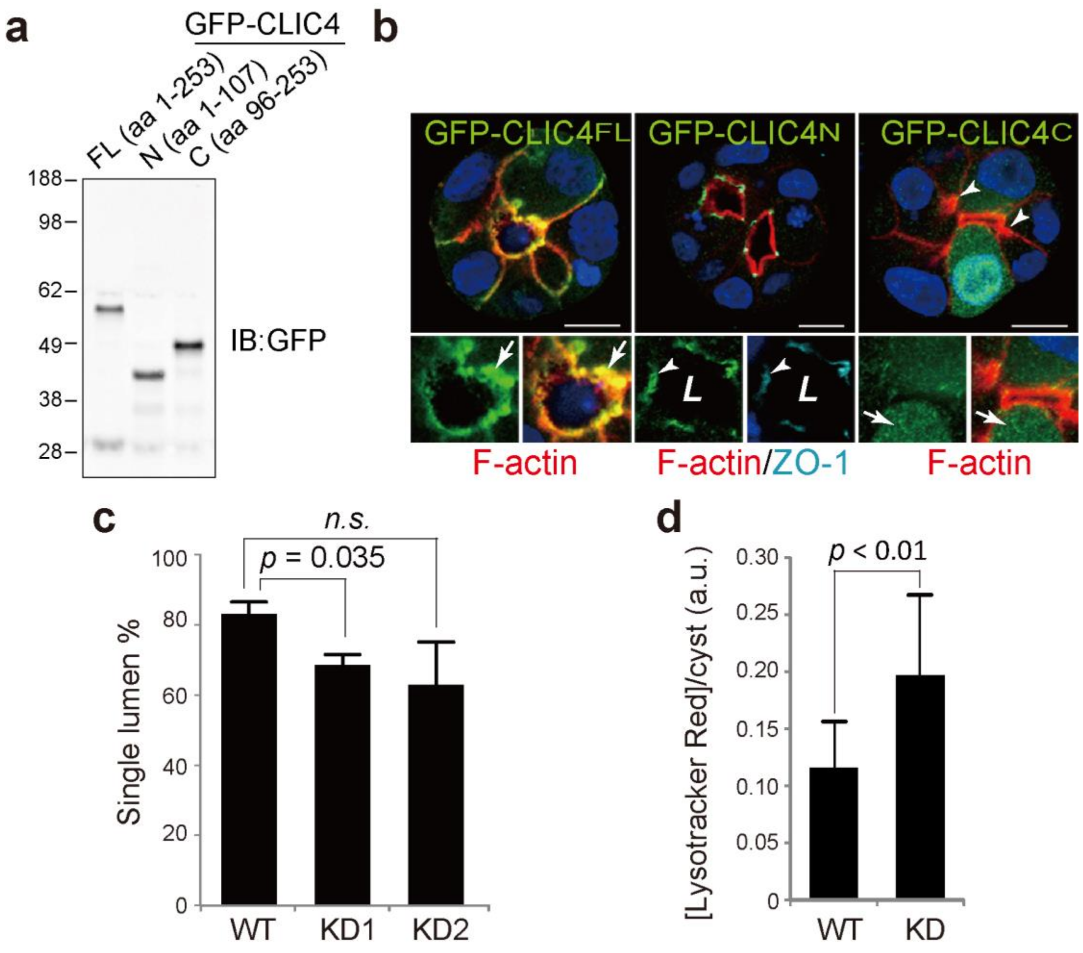

Supplementary Figure 5 Expression of CLIC4 variants in MDCK cysts

(**a**) Immunoblots of MDCK lysates expressing three indicated GFP fusion proteins probed with anti-GFP antibody. The predominant bands match their predicted molecular mass respectively. FL (full- length): aa1-253; N: aa1-107; C:aa96-253.

(**b**) 3-day cyst cultures formed by CLIC4-KD cells transfected with indicated GFP fusions were labeled for F-actin or together with ZO-1. Arrows in the left panel point to the enrichment of the full-length CLIC4 and F-actin at the central lumen. Arrowheads in the middle panel points to the co-distribution of GFP-CLIC4$_N$ and ZO-1 in the TJs of an ectopic lumen. Arrows and arrowheads in the right panel point to the cytosolic GFP-CLIC4$_C$ and two ectopic lumens, respectively. L: lumen. Scale bars = 10 μm.

(**c**) Quantification of single lumen-containing 6-day cysts. *p* value by *t*-test; n > 240; 3 repeats. Error bars represent standard deviation.

(**d**) To quantify lysosomes, the 3-day cysts were incubated with 1 μM LysoTracker® Red DND-99 (Invitrogen) for 20 min at 37°C. After three times washes, the stained cysts were imaged in the recording buffer (Hank's balanced salt solution supplemented with 1% FBS and 4.5 g/L glucose). The overall signal intensity of an individual cyst was summed up from 4 center z-sections (3 μm-thick). The intensity was further normalized by the area size of the given cyst. *p* value by *t* test, n = 21 cysts for WT; n = 30 cysts for CLIC4-KD. Error bars present for standard deviation.

.

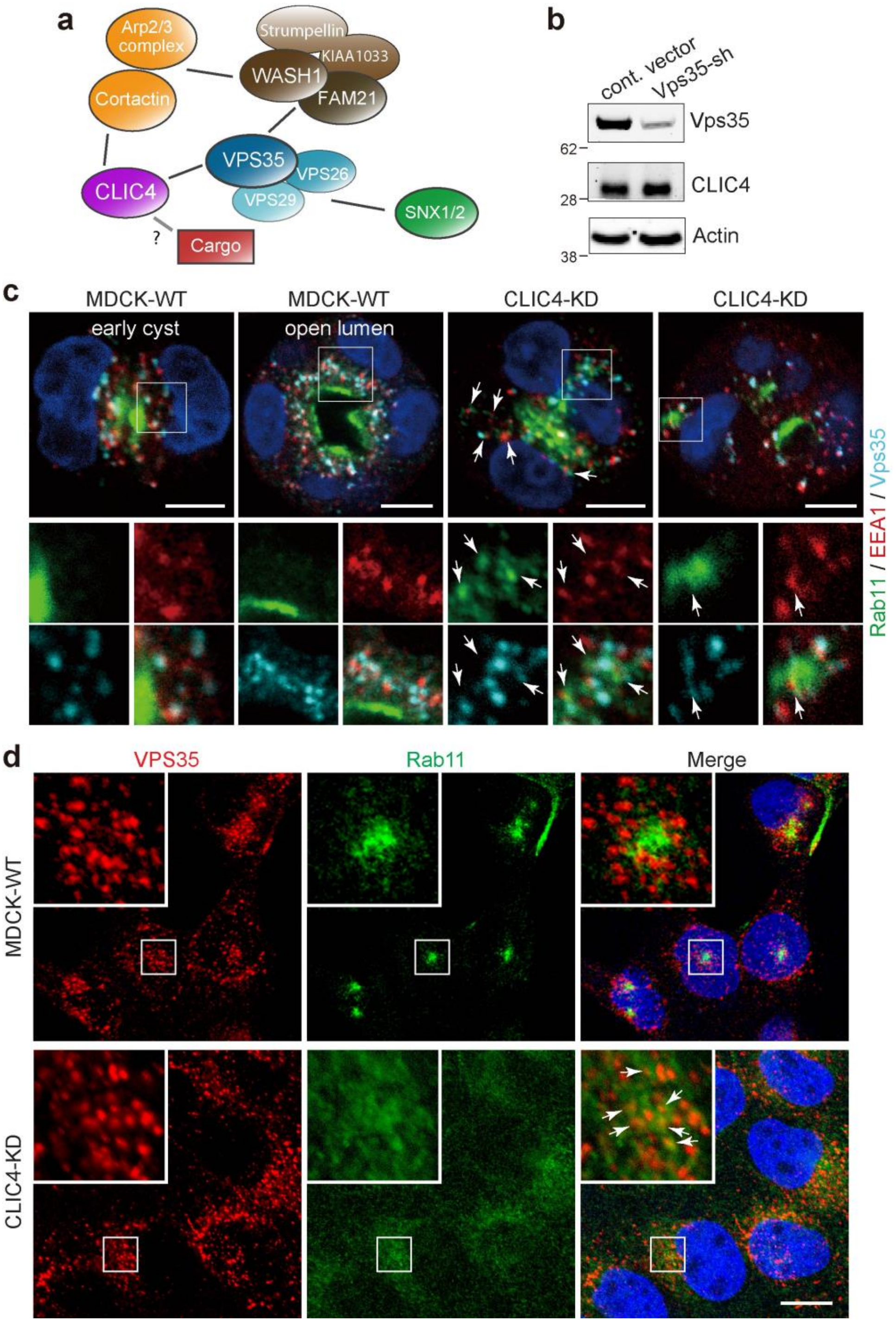

Supplementary Figure 6 CLIC4 depletion results in disordering of the endolysosomal system
(**a**) A diagram depicting the interrelationship of the retromer subcomplex (shown in blue), the sorting nexin complex (shown in green), the WASH1 complex (shown in brown), Arp2/3 complex (shown in orange) and cortactin on EE surface. Black lines indicate the previously reported protein-protein physical link. Here we report the CLIC4-Vps35 and CLIC4-cortactin interaction; whether CLIC4 also directly interacts with cargos remains unclear.
(**b**) Protein blots of MDCK cells transfected with control vector, or Vps35-shRNA (for 3 days) were probed with indicated antibodies.

(**c**) 3D cysts, formed by WT or mutant MDCK cells with CLIC4 inducibly suppressed, were immunolabeled at different stages as indicated (1-day for early cyst and 3-day for open lumen). Enlarged views of boxed areas are shown to highlight the close association between Vps35 and EEA1 in WT cysts, as well the spatial segregation between EEA1-labeled EEs and Rab11a-labeled REs in more mature cysts that had an open lumen. By contrast, the physical separation between EEA1/Vps35, and Rab11a signals was impaired in the CLIC4-KD cysts; many compartments had both Rab11a and EEA1 (or Vps35) (arrows).
(**d**) Double labeling of Vps35 and Rab11a of subconfluent MDCK cultures. The pericentriolar Rab11a-positive REs were largely devoid of the Vps35 signal in the WT cells. In CLIC4-KD mutant cells, the perinuclear enrichment of Rab11a was lost in the CLIC4-KD mutant cells; instead, Rab11a signal was distributed diffusely throughout the cells and closely associated with Vps35 labeling (arrows).
Scale bars = 10 μm (c, d).

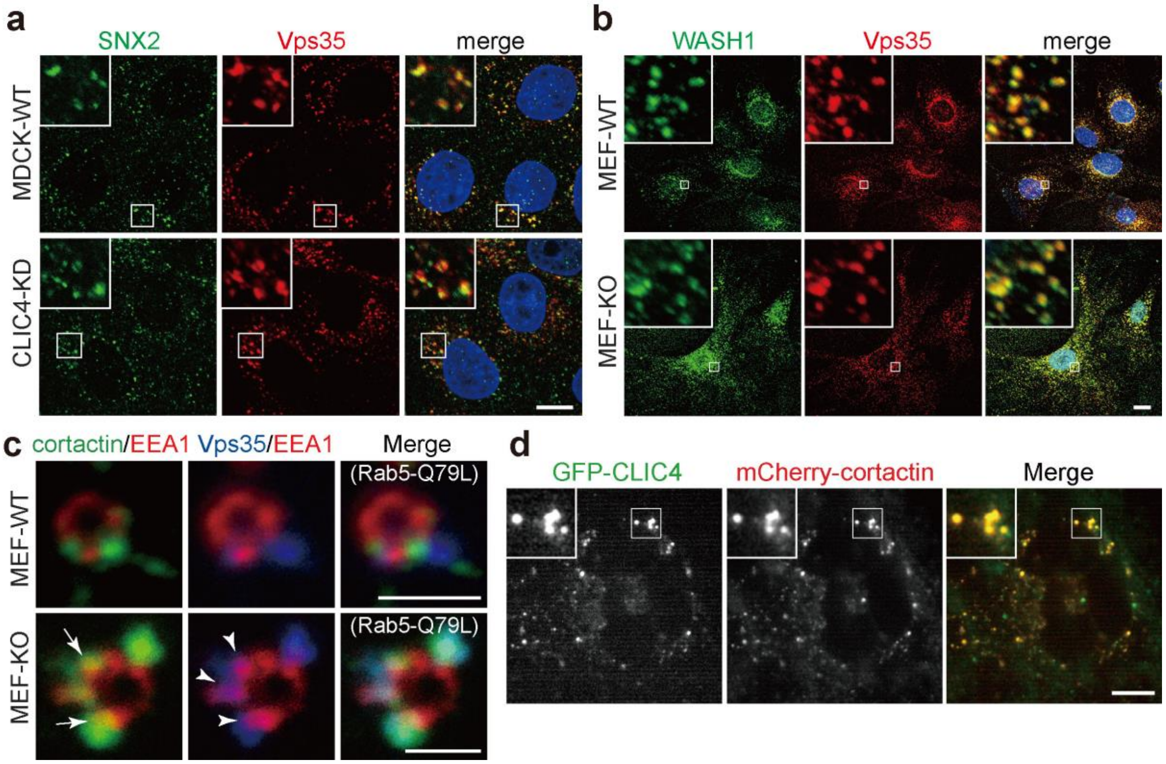

Supplementary Figure 7 CLIC4 has minimal effect on EE recruitment of retromer

(**a, b**) Immunostaining of Vps35 and SNX2 in MDCK cells (a) or WASH1 in MEFs (b). Enlarged views of the boxed areas are shown. Pearson's coefficient = 0.34±0.10 (Vps35 vs. SNX2 in WT, n = 10), 0.51± 0.08 (Vps35 vs. SNX2 in KD, n = 10); 0.40±0.06 (Vps35 vs. WASH1 in WT, n = 10), 0.43±0.12 (Vps35 vs. WASH1 in KO, n = 10).

(**c**) WT and CLIC4-KO MEFs transfected with Flag-Rab5Q79L. Representative individual "swollen" EE triply labeled for cortactin, Vps35, and EEA1 are shown. In the CLIC4-KO MEFs, EEA1 often overlapped with cortactin (arrows) and Vps35 (arrowheads).

(**d**) A representative frame of live images taken from a transfected MEF expressing low levels of GFP-CLIC4 and mCherry-cortactin. Enlarged views of the boxed area demonstrate the extensive colocalization of these two molecules. Pearson's coefficient = 0.77±0.11 (n = 6).

Scale bars = 10 μm (a, b, d); 2 μm (c).

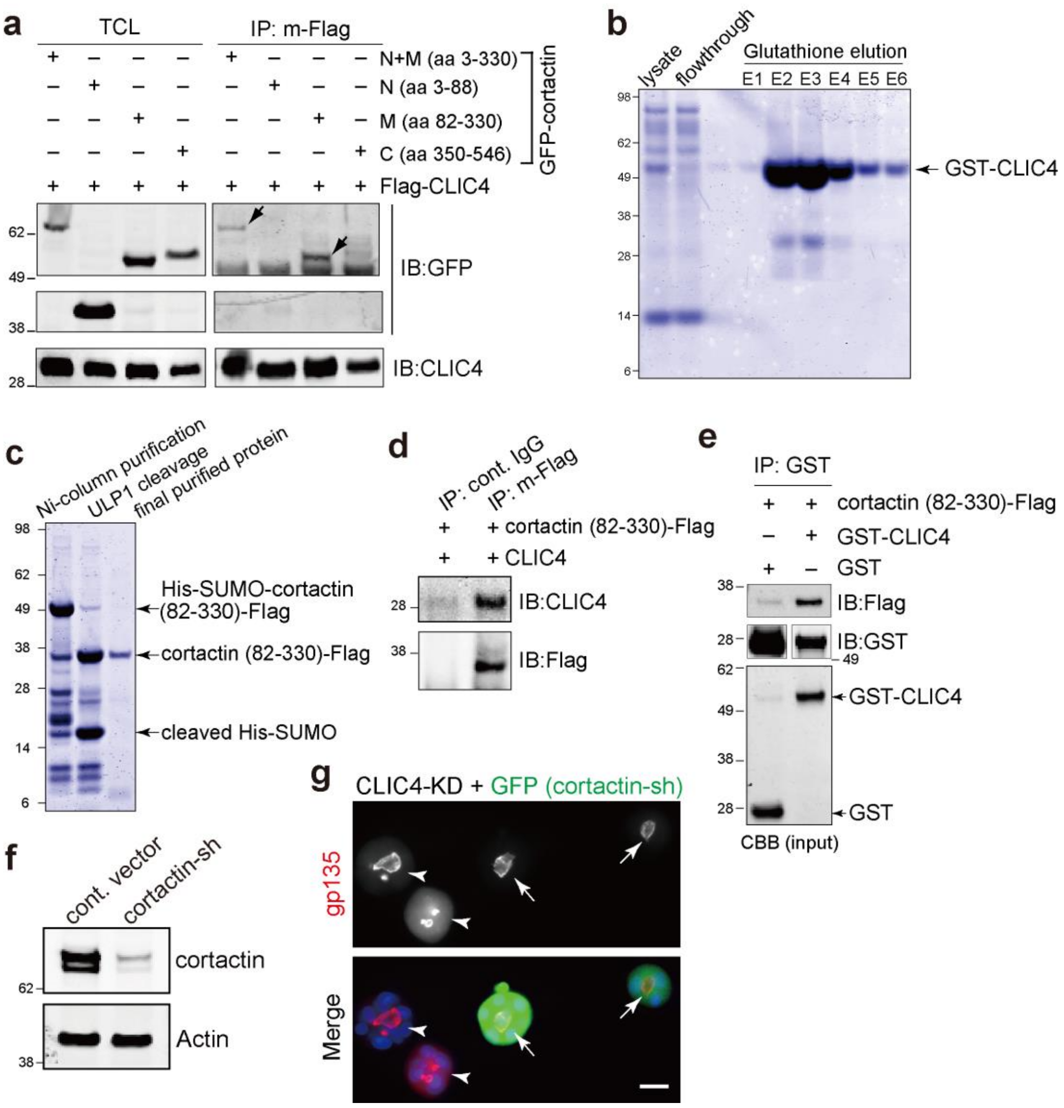

Supplementary Figure 8 Cortactin-CLIC4 interaction and cortactin-shRNA effect on luminogenesis

(**a**) Total cell lysates (TCL) and Flag-antibody immunoprecipitates (IP) of HEK cells transfected with Flag-CLIC4 together with various GFP-cortactin truncates were subject to immunoblot with anti-GFP and anti-CLIC4 antibodies. Arrows point to the GFP-cortactin fragments co-precipitated with Flag-CLIC4. Note that the ~50 kDa bands appeared in all the lanes,were IgG heavy chains.

(**b**) Purification of GST-CLIC4 recombinant protein. *BL21* bacterial lysates expressing GST-CLIC4 were passed through glutathione sepharose 4B (GH Healthcare) and eluted with 20 mM reduced glutathione. Samples from each step were analyzed by SDS-PAGE and visualized by Coomassie Blue staining.

(**c**) Purification of Flag-tagged cortactin(82-330) recombinant protein. *BL21* lysates expressing His_6-SUMO-cortactin(82-330)-FLAG were first purified by Ni-NTA agarose column (Qiagen), cleaved by ULP1 protease to remove the His_6-SUMO tag [3], and then subjected to the second round of Ni-NTA column purification. The final cortactin fragment appears as a single protein band of expected molecular mass as seen on the SDS-PAGE stained with Coomassie blue.

(**d**) Pull down assays. The mixture of purified cortactin (82-330)-Flag and CLIC4 peptides were subjected to immuno-pull down with either an anti-Flag M2 antibody or the species-matched mouse IgG (cont.). The resulting immunoprecipitates were probed with indicated antibodies.

(**e**) Reciprocal pull down assays using an anti-GST antibody. The immunoblots of immunoprecipates probed by indicated antibodies are shown. A Coomassie Blue (CBB) stained gel was shown to demonstrate that roughly equal amounts of input (GST, GST-CLIC4) were employed.
(**f**) MDCK cells transfected with control vector or cortactin-shRNA for 3 days were harvested for immunoblotting with indicated antibodies.
(**g**) Immunolabeling of gp135 and GFP of 2.5-day cysts formed by CLIC4-KD MDCK cells transiently transfected with a vector expressing both cortactin-shRNA and GFP. Untransfected, GFP-negative CLIC4-KD cysts developed multilumens (arrowheads), whereas GFP+ transfected CLIC4-KD cysts formed a single lumen (arrows). Scale bar = 20 μm.

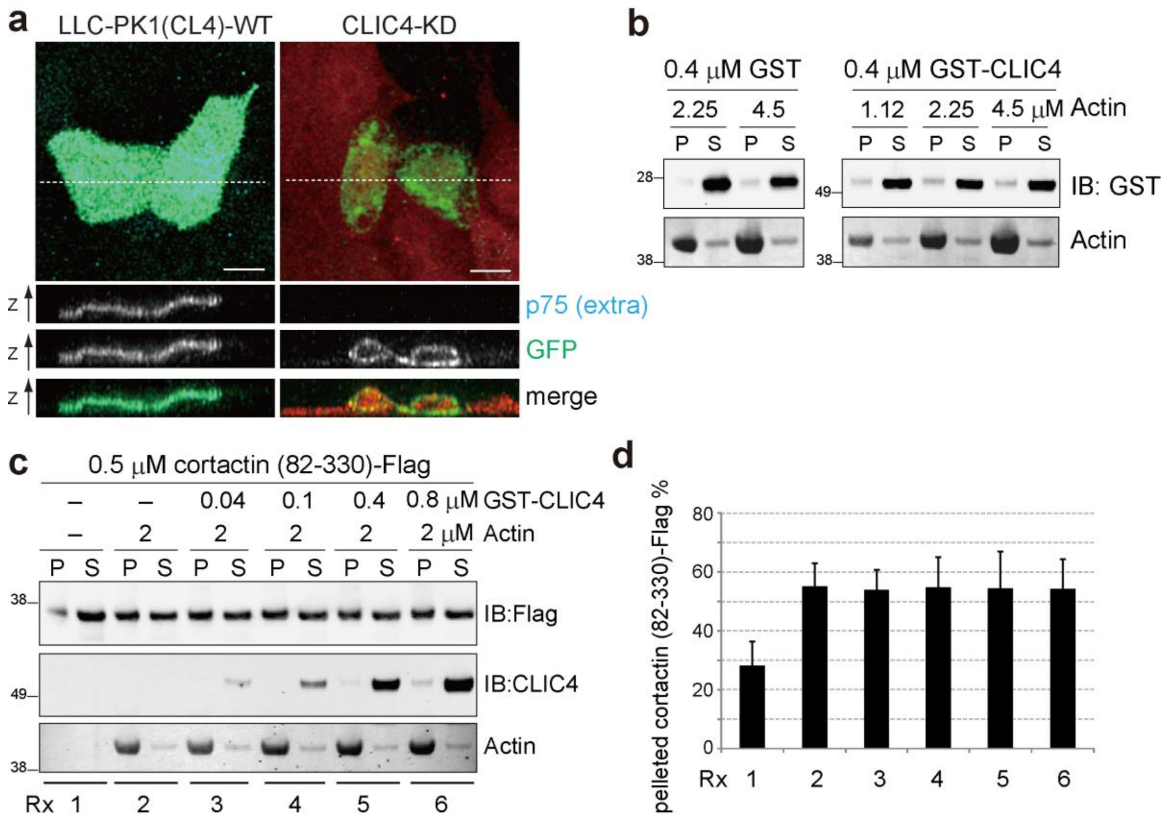

Supplementary Figure 9 CLIC4 affects p75 trafficking in LLC-PK1 and F-actin cosedimentation
(**a**) LLC-PK1(CL4) cells expressing CLIC4-shRNA/RFP (KD) for 4 days were transfected with p75-GFP for 1 day. Cells were then fixed and stained with an anti-p75 antibody specifically recognizing its extracellular domain in a non-permeabilized condition. Both x-y (upper panels) and x-z (lower three panels) confocal views are shown; arrows point to the apical side. Note that in WT cells, the staining signals of p75 and GFP were extensively overlapped on the apical surface. In contrast, in CLIC4-KD cells p75-GFP was undetectable on the apical surface; GFP signals appeared intracellularly. Scale bars = 10 μm.

(**b**-**d**) *In vitro* F-actin cosedimentation assays [4]. Actin subunits (>99% pure; Cytoskeleton, cat# AKL99) were polymerized according to company manual. Recombinant cortactin(82-330)-Flag and various amounts of GST-CLIC4 (Rxs 1-6) were incubated with polymerized F-actin (total volume 100 μl) for 1 h and then spun down at 100,000 *x g* for 1h. Equivalent fractions of supernatant (S) and pellet (P) were analyzed by SDS-PAGE and followed by immunoblotting (IB) or ponceau S staining (for actin). (d) represents a quantification of the assays described in (c). The percentage of pelleted cortactin was calculated by the formula: (P/P+S) x100. Results were from 3 independent repeats. Error bars = standard deviation.

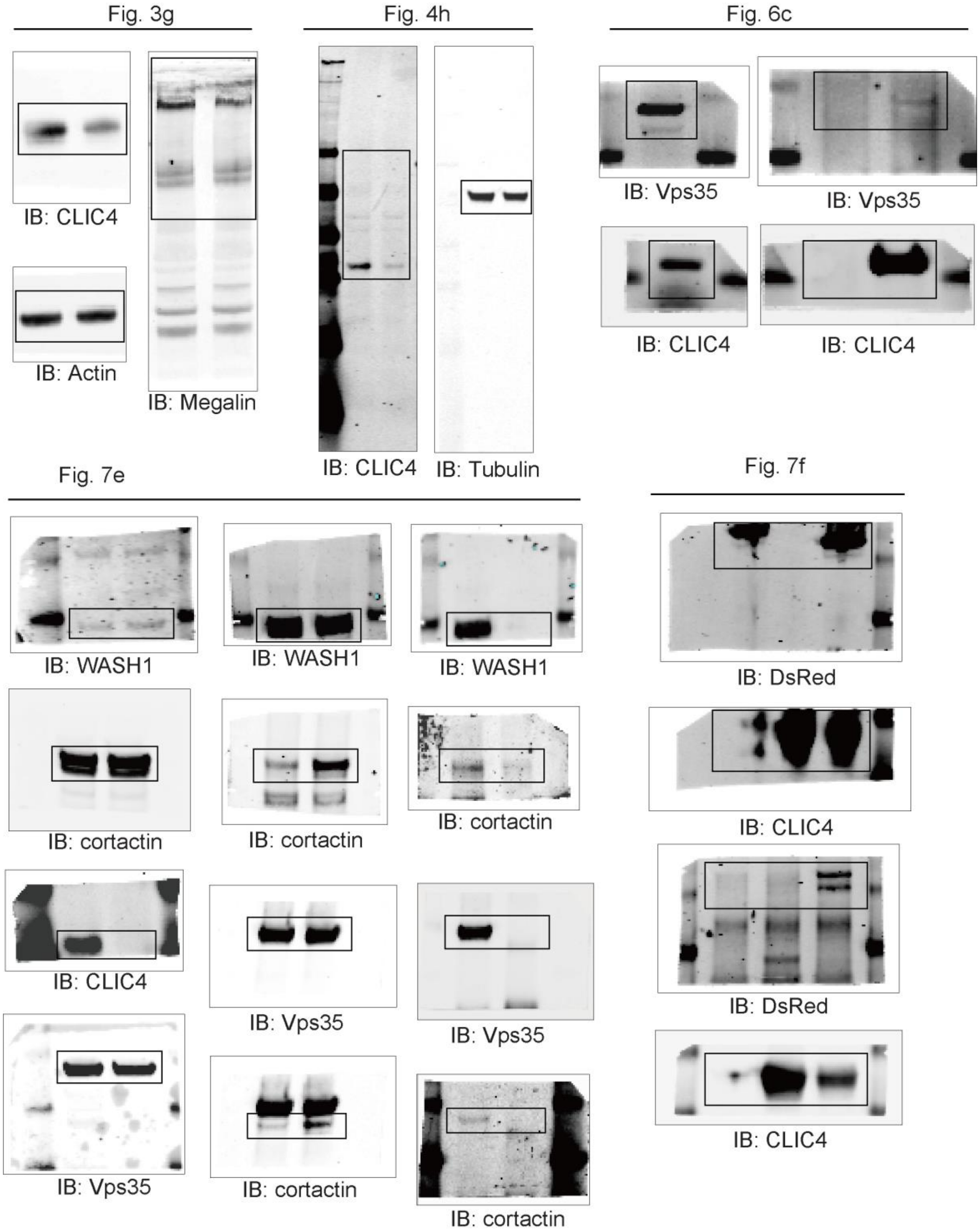

Supplementary Figure 10 Original immunoblotting and gel images

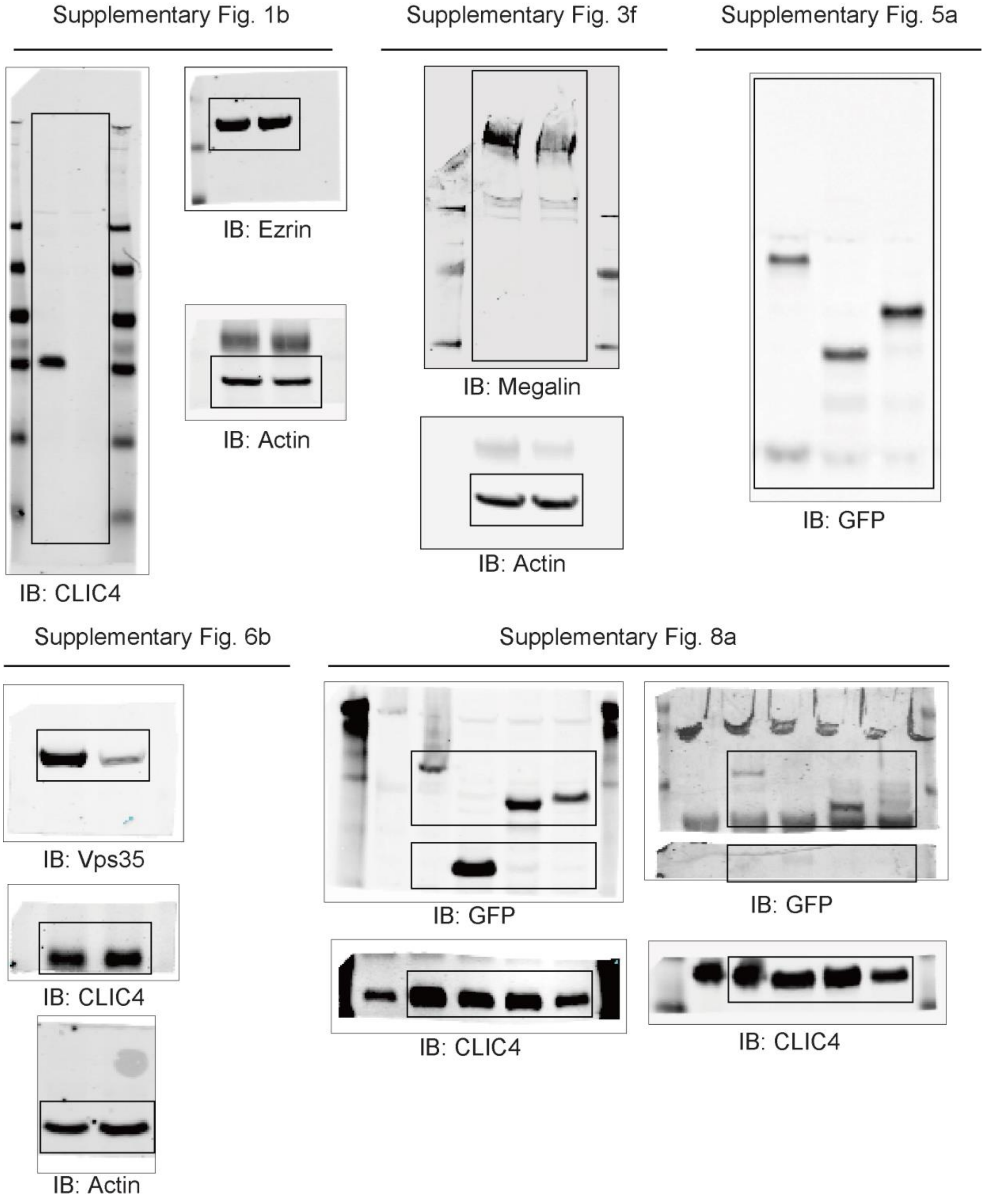

Supplementary Figure 10. (continued)

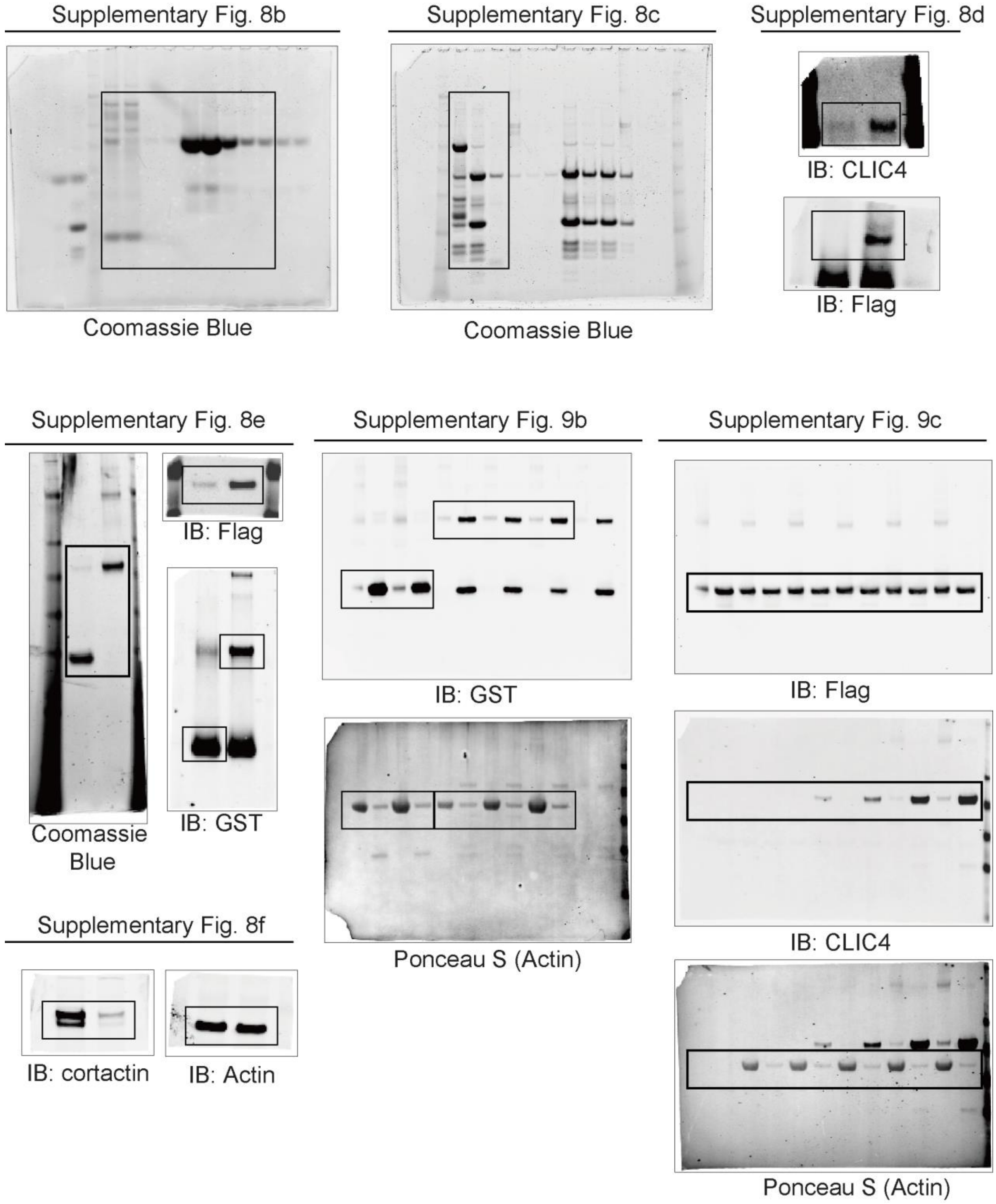

Supplementary Figure 10. (continued)

Supplementary Note 1

We confirmed CLIC4 immunolabeling specificity using several methods. First, the characteristic CLIC4 labeling pattern was largely diminished when the CLIC4 antibody was pre-absorbed by the CLIC4-maltose binding protein fusion, but not by maltose binding protein alone (Supplementary Figure 1a). Second, CLIC4 antibody recognized a single band of expected size in renal lysates of WT mice (Supplementary Figure 1b). This protein band, but not the control proteins, ezrin and actin, were specifically lost in renal lysates of CLIC4-KO mice (Supplementary Figure 1b). Third, CLIC4 immunolabeling largely disappeared in CLIC4-KO mouse kidney sections (Figs. 1h,i). Loss of CLIC-4 protein in lysates, and CLIC-4 staining in kidney sections, is consistent with our previous report demonstrating depletion of the *clic4* gene in these CLIC4-KO mice [5]. Finally, our previous characterization showed that our CLIC4 antibody does not cross react with several other CLIC proteins [1].

Supplementary Note 2

There are several possibilities that may explain why MV membrane specialization could be related to PT lumen size control. First, the convoluted MV membranes provide a large surface area to house the ion exchangers and/or water channels (e.g., NHE3) for active fluid transport regulation. The decreased expression of these molecules as a consequence of reduced overall apical surfaces could contribute to PT dilation.

Second, since megalin is essential for renal PT reabsorption [6] the decreased expression of megalin might further compromise the reabsorption ability of CLIC4-KO PT cells. Mutations in the human megalin gene are responsible for Donnai-Barrow and Facio-Oculo-Acoustico-Renal syndromes [7]. Interestingly, these patients not only feature PT dysfunction (e.g., proteinuria) but also retinal detachment. Retinal detachment (caused by excess fluid accumulated in the luminal/subretinal space between the RPE and neural retina) is also a prominent phenotype seen in rats with CLIC4 suppressed in RPE cells [8].

Third, while little is known in mammals, proper secretion of glycan-containing proteins into the lumen critically determines the diameter and/or shape of the lumen in fly hindgut tube, trachea, and eyes and the excretory canal of C. elegans [9-14]. The abnormal staining pattern of PAS and LTA (both of which recognize glycoproteins) and the altered electron density of luminal cavity indicate that CLIC4-KO kidneys had defects in luminal secretion and/or remodeling of glycan-containing ECM. The sugar-modified macromolecules may regulate the tube structure over a long distance via scaffolding, electrostatic repulsion, and/or mechanical forces. Plausibly, the altered luminal secretion of ECM components could also contribute to the dilation of CLIC4-KO PT.

Supplementary Methods

Reagents and antibodies

All chemicals were purchased from Sigma unless otherwise indicated. Antibodies and dilutions for IB (immunoblotting) or IFA (immunostaining) were as followed: actin (mouse, Sigma; IB: 1:250), β-catenin (mouse, BD Bioscience; IFA: 1:200), CI-MPR (mouse, Abcam; IFA: 1:100), CLIC4 (rabbit [1], made by our lab; IFA: 1:100 and IB: 1:1000), cortactin (mouse, Millipore; IFA: 1:200 and IB: 1:1000), cytokeratin (mouse; Sigma; IFA: 1:100), DsRed (rabbit, Clontech; IB: 1:1000), EEA1 (mouse, BD Bioscience; goat, Santa Cruz; rabbit, Cell Signaling; all for IFA: 1:200), Ezrin (mouse, Abcam; IFA:1:200 and IB: 1:1000), Flag (mouse clone M2, Sigma; IFA: 1:1000 and IB: 1:2500), GFP (chicken, Abcam; IFA: 1:1000 and IB: 1:2000), GFP (mouse, Sigma; IFA: 1:1000 and IB: 1:2000), gp80 (rabbit; a gift from Dr. Claudia Koch-Brandt [15]; IFA: 1:200), gp135 (mouse clone 3F2/D8, a gift from Dr. George Ojakian; IFA: 1:200), gp135 (rabbit, a gift from Tzuu-Shuh Jou; IFA: 1:200), GST (rabbit, made by our lab; IB: 1:4000), LAMP1 (mouse clone AC17, DHSB; IFA: 1:10), mCherry (goat, Sicgen; IFA: 1:600), megalin (rabbit, a gift from Dr. Daniel Biemesderfer [16]; IFA: 1:600 and IB: 1:2000), Na,K-ATPase (mouse, DHSB; IFA: 1:150), NHE3 (rabbit, Chemicon; IFA: 1:200), P75 (mouse, a gift from Anne Muesch; IFA: 1:300), Par3 (rabbit, a gift of Dr. Songhai Shi [17]; IFA: 1:300), PKCλ (mouse, BD Bioscience; IFA:1:400), PTEN (rabbit, R&D Systems; IFA: 1:600), Rab11a (rabbit, Invitrogen; IFA: 1:200), Snx2 (mouse, BD Biosceince; IFA: 1:200), α-Tubulin (mouse, Sigma; IB: 1:2000), Vps35 (goat, Abnova; IFA:1:200 and IB: 1:1000), WASH1 (rabbit, Millipore and a gift from Dr. Daniel Billadeau [18]; IFA: 1:1000 and IB: 1:2000), ZO-1 (rat, a gift from Dr. Enrique Rodriguez-Boulan; IFA: 1:2000), Alexa-dye conjugated secondary antibodies (Invitrogen; IFA: 1:400), Alexa-dye conjugated streptavidin (Invitrogen, IFA: 1:1000), Alexa-dye conjugated phalloidin (Molecular Probes, IFA: 1:400), Biotinylated LTA (Vector Labs, IFA: 1:4000), and Ultrasmall gold-conjugated secondary antibodies (EMS; 1:50).

The shRNA sequence targeting canine CLIC4 was 5'-CCACTACTTATTATAGTAAT-3'. The $CLIC4_d$-shRNA (under U6 promoter) was further inserted into a vector containing the neomycin resistance gene and an inducible GFP cassette that was flanked by loxP to result in $CLIC4_d$-sh-neo-loxP-GFP-loxP vector. The short hairpin RNA sequence against cortactin was 5'-GGGTGCAGAAGGATCGGATGGA-3'. Vps29-mCherry and Vps35-shRNA/GFP were kind gifts of Mark von Zastrow [19]. The plasmid encoding GFP-Rab11a was generated by inserting the EcoRI/XhoI fragment of Rab11a in pcDNA3 [20] into EcoRI/SalI digested pEGFP-C2 vector (Clontech). For generating N-terminal GFP tagged human CLIC4 truncated variants, the N- (from Met^1 to Leu^{107}) and C-terminal (from Glu^{96} to Lys^{253}) DNA fragments of human CLIC4 were amplified by standard PCR method and subcloned into pEGFP-C1 vector (Clontech). Cdc42-WT-HA, Cdc42-G12V-HA, and Cdc42-T17N-HA were purchased from Gutheria. Flag-Rab5 [21], GFP-Rab7 (gift of Dr. Colin Parrish, [22]), Flag-Rab8, Flag-Rab11a (a gift from Dr. Tim McGraw [20]), GFP-Cdc42 [23], mCherry-LifeAct (a gift from Dr. Guillaume Montagnac [24]), GFP fusions of human CLIC4 (a gift from Mark Berryman), canine CLIC4 [8], mCherry-(mouse) cortactin (a gift from Christien Merrifield [25]; Addgene plasmid #27676), and the mCherry-Rab11a expressing MDCK stable line [26] have been previously reported. GFP-(mouse) cortactin in pCAG vector was generated by inserting BsrG/BamHI-cut, PCR fragment of full-length mouse cortactin cDNA (a gift from Anna Huttenlocher [27]; Addgene plasmid #26722) into BsrGI/BglII-digested vector. Plasmids encoding cortactin truncates were generated by PCR amplification of the fragment encoding N (K^3 to F^{88}), N+M (from K^3 to V^{330}), M (from H^{82} to V^{330}), or C (from I^{350} to Q^{546}) region of mouse cortactin using the following primer pairs followed by standard molecular cloning method. N (5'-CATGGTCCTGCTGGAGTTCGTG-3' and 5'-TGATAGGGATCCTACACCTGGACCACTTCTTC-3'), N + M (5'-CATGGTCCTGCTGGAGTTCGTG-3' and 5'-

CCGAACGGATCCTAATAGCCGTGGGAAGCC-3'), M (5'-CTTCCCTGTACAAGCACGGCTATGGCGGGA -3' and 5'-TGATAGGGATCCTACACCTGGACCACTTCTTC-3'), or C (5'-GCAAAATGTACAAGATCCGTGCTAACTTTG -3' and 5'-TCCCATATGTCCTTCCGAGTG -3'). To generate the bacterial expression construct encoding His_6-SUMO-cortactin-FLAG fusion protein, the coding region of mouse cortactin (amino acids, 82 to 330) and a C-terminal Flag tag was PCR amplified (5'-CCACGGCTATGGCGGGAAG-3' and 5'-TCGAGTTACTTATCGTCATCGTCTTTGTAGTCCACCTGGACCACTTCTTCAAAGG-3'). The PCR fragment was annealed with another PCR product amplified by 5'-GATCCCACGGCTATGGCGGGAAG-3' and 5'-GTTACTTATCGTCATCGTCTTTGTAGTCC-3', for generating the sticky ends of *BamH*I and *Xho*I, and cloned into pSMT3 vector (gift of Neal Lue).

Generation of CLIC4-KO mice and CLIC4-KO MEFs

All animal manipulations were performed in accordance with the IACUC guidelines for animal experiments at Weill Cornell Medical College. The generation of CLIC4-floxed homozygotes (*clic4*$^{f/f}$) and CLIC4-KO mice (both are in a pure C57BL/6J background) as well as the genotyping methods have been previously described [5]. To avoid the putative cytotoxicity caused by constitutive expression of Cre recombinase [28], we used exclusively Cre-negative, CLIC4 null mice in these studies. Briefly, germline-transmitted heterozygous floxed mice CLIC4$^{flox/+}$ mice (in C57BL6/J background) were bred with CAG-Cre mice (a universal deleter in C57BL/6J background) to generate heterozygous KO (*clic*4$^{+/-}$) mice. Some of the female heterozygotes were Cre-negative (*cre*$^{-}$;*clic4*$^{+/-}$), consistent with previous studies showing that mature oocytes of CAG-Cre transgenic females contain sufficient Cre activity to mediate the deletion of paternally derived LoxP-flanked DNA sequences upon fertilization irrespective of the transmission of Cre transgene [29]. These female mice were mated with C57BL/6J mice to generate Cre-negative heterozygous *clic4* deleted male and female mice (*cre*$^{-}$;*clic4*$^{+/-}$), which were subsequently mated to each other to generate homozygous *cre*$^{-}$; *clic4*$^{-/-}$ mice. We used homozygous Cre-negative CLIC4 KO mice (*cre*$^{-}$;*clic4*$^{-/-}$) and their WT littermates for comparison. To obtain mouse embryonic fibroblast cells, E13.5 embryos were finely minced and incubated with trypsin (0.125%)-EDTA and DNase (4 mg/ml) in PBS with gentle agitation at 37°C for 15 minutes. After gentle pipetting, the tissue digests were diluted with DMEM and 10% FBS, followed by 1000 rpm for 5 minutes. The cell pellets were resuspended and maintained in DMEM plus 10% FBS until cells reached confluence before passaging and frozen storage. Note that two additional CLIC4-KO mouse lines have been previously described [30] [31].and were generated using different targeting alleles in different genetic backgrounds.

Generation of MDCK and LLC-PK1 stable lines

A LLC-PK1 clone CL4 (previously reported to have longer MV, gift from Dr. James R. Bartles [32]) was maintained in alpha minimum essential medium (GIBCO) containing 5% FBS (Gemini) as described previously [33]. MDCK cells were maintained in DMEM medium (Cellgro) containing 5% FBS. Stable clones expressing GFP fusion of canine CLIC4 were selected by G418 resistance; only clones that expressed modest levels of GFP were chosen for analysis. To generate stable clones with CLIC4 constitutively suppressed, MDCK cells were transfected with plasmid encoding CLIC4 (canine) shRNA-neo-loxP-GFP-loxP and followed by G418 selection. The surviving clones were infected with adenovirus encoding Cre recombinase (Vector Development Lab); the GFP+ clones were further validated for CLIC4 reduction on immunoblots. Results presented here are representative examples of two independent clones.

Lentiviruses encoding both doxycycline-regulated shRNA against CLIC4 and turboRFP (Thermo Scientific, V3THS_376175: 5'-GTCATTTCATTGCCATCCA-3') were also used to infect MDCK or

LLC-PK1(CL4) cells for either transiently expressing CLIC4-sh/RFP or establishing stable clones followed by puromycin selection. Doxycycline (0.4 μg/ml) was added into the medium for 3 days before plating for staining, 3D culture, or other biochemical analyses. Note that the parental LLC-PK1(CL4) we obtained [32] had heterogeneous megalin expression. To facilitate our investigation, we chose LLC-PK1(CL4) stable clones that had homogenous expression of megalin and effective doxycycline-induced CLIC4 silencing for further analysis.

SUPPLEMENTARY REFERENCES

1 Chuang, J. Z., Milner, T. A., Zhu, M. & Sung, C. H. A 29 kDa intracellular chloride channel p64H1 is associated with large dense-core vesicles in rat hippocampal neurons. *J Neurosci* **19**, 2919-2928 (1999).
2 Kalakeche, R. *et al.* Endotoxin uptake by S1 proximal tubular segment causes oxidative stress in the downstream S2 segment. *J Am Soc Nephrol* **22**, 1505-1516 (2011).
3 Steinberg-Neifach, O., Wellington, K., Vazquez, L. & Lue, N. F. Combinatorial recognition of a complex telomere repeat sequence by the Candida parapsilosis Cdc13AB heterodimer. *Nuclei Aci Res* **43**, 2164-2176 (2015).
4 Weed, S. A. *et al.* Cortactin localization to sites of actin assembly in lamellipodia requires interactions with F-actin and the Arp2/3 complex. *J Cell Biol* **151**, 29-40 (2000).
5 He, G. *et al.* Role of CLIC4 in the host innate responses to bacterial lipopolysaccharide. *Eur J Immunol* **41**, 1221-1230 (2011).
6 Verroust, P. J., Birn, H., Nielsen, R., Kozyraki, R. & Christensen, E. I. The tandem endocytic receptors megalin and cubilin are important proteins in renal pathology. *Kidney Int* **62**, 745-756 (2002).
7 Kantarci, S. *et al.* Mutations in LRP2, which encodes the multiligand receptor megalin, cause Donnai-Barrow and facio-oculo-acoustico-renal syndromes. *Nat Genet* **39**, 957-959 (2007).
8 Chuang, J. Z., Chou, S. Y. & Sung, C. H. Chloride Intracellular channel 4 Is critical for the epithelial morphogenesis of RPE cells and retinal attachment. *Mol Biol Cell* **21**, 3017-3028 (2010).
9 Devine, W. P. *et al.* Requirement for chitin biosynthesis in epithelial tube morphogenesis. *Proc Natl Acad Sci U S A* **102**, 17014-17019 (2005).
10 Syed, Z. A. *et al.* A luminal glycoprotein drives dose-dependent diameter expansion of the Drosophila melanogaster hindgut tube. *PLoS Genet* **8**, e1002850 (2012).
11 Husain, N. *et al.* The agrin/perlecan-related protein eyes shut is essential for epithelial lumen formation in the Drosophila retina. *Dev Cell* **11**, 483-493 (2006).
12 Luschnig, S., Batz, T., Armbruster, K. & Krasnow, M. A. serpentine and vermiform encode matrix proteins with chitin binding and deacetylation domains that limit tracheal tube length in Drosophila. *Curr Biol* **16**, 186-194 (2006).
13 Luschnig, S. & Uv, A. Luminal matrices: An inside view on organ morphogenesis. *Exp Cell Res* **321**, 64-70 (2013).
14 Jones, S. J. & Baillie, D. L. Characterization of the let-653 gene in Caenorhabditis elegans. *Mol Gen Genet* **248**, 719-726 (1995).
15 Urban, J., Parczyk, K., Leutz, A., Kayne, M. & Kondor-Koch, C. Constitutive apical secretion of an 80-kD sulfated glycoprotein complex in the polarized epithelial Madin-Darby canine kidney cell line. *J Cell Biol* **105**, 2735-2743 (1987).
16 Biemesderfer, D., DeGray, B. & Aronson, P. S. Active (9.6 s) and inactive (21 s) oligomers of NHE3 in microdomains of the renal brush border. *J Biol Chem* **276**, 10161-10167 (2001).
17 Bultje, R. S. *et al.* Mammalian Par3 regulates progenitor cell asymmetric division via notch signaling in the developing neocortex. *Neuron* **63**, 189-202 (2009).
18 Gomez, T. S. & Billadeau, D. D. A FAM21-containing WASH complex regulates retromer-dependent sorting. *Dev Cell* **17**, 699-711 (2009).
19 Choy, R. W. *et al.* Retromer mediates a discrete route of local membrane delivery to dendrites. *Neuron* **82**, 55-62 (2014).
20 Zeigerer, A. *et al.* GLUT4 retention in adipocytes requires two intracellular insulin-regulated transport steps. *Mol Biol Cell* **13**, 2421-2435 (2002).
21 Hu, Y., Chuang, J. Z., Xu, K., McGraw, T. E. & Sung, C. H. SARA, a FYVE domain protein, affects Rab5-mediated endocytosis. *J Cell Sci* **115**, 4755-4763 (2002).
22 Harbison, C. E., Lyi, S. M., Weichert, W. S. & Parrish, C. R. Early steps in cell infection by parvoviruses: host-specific differences in cell receptor binding but similar endosomal trafficking. *J Virol* **83**, 10504-10514 (2009).

23 Nalbant, P., Hodgson, L., Kraynov, V., Toutchkine, A. & Hahn, K. M. Activation of endogenous Cdc42 visualized in living cells. *Science* **305**, 1615-1619 (2004).
24 Lizarraga, F. *et al.* Diaphanous-related formins are required for invadopodia formation and invasion of breast tumor cells. *Cancer Res* **69**, 2792-2800 (2009).
25 Taylor, M. J., Perrais, D. & Merrifield, C. J. A high precision survey of the molecular dynamics of mammalian clathrin-mediated endocytosis. *PLoS Biol* **9**, e1000604 (2011).
26 Thuenauer, R. *et al.* Four-dimensional live imaging of apical biosynthetic trafficking reveals a post-Golgi sorting role of apical endosomal intermediates. *Proc Natl Acad Sci U S A* **111**, 4127-4132 (2014).
27 Perrin, B. J., Amann, K. J. & Huttenlocher, A. Proteolysis of cortactin by calpain regulates membrane protrusion during cell migration. *Mol Biol Cell* **17**, 239-250 (2006).
28 Schmidt-Supprian, M., Wunderlich, F. T. & Rajewsky, K. Excision of the Frt-flanked neo (R) cassette from the CD19cre knock-in transgene reduces Cre-mediated recombination. *Transgenic Res* **16**, 657-660 (2007).
29 Sakai, K. & Miyazaki, J. A transgenic mouse line that retains Cre recombinase activity in mature oocytes irrespective of the cre transgene transmission. *Biochem Biophys Res Commun* **237**, 318-324 (1997).
30 Padmakumar, V. C. *et al.* Spontaneous skin erosions and reduced skin and corneal wound healing characterize CLIC4(NULL) mice. *Am J Pathol* **181**, 74-84 (2012).
31 Ulmasov, B., Bruno, J., Gordon, N., Hartnett, M. E. & Edwards, J. C. Chloride intracellular channel protein-4 functions in angiogenesis by supporting acidification of vacuoles along the intracellular tubulogenic pathway. *Am J Pathol* **174**, 1084-1096 (2009).
32 Zheng, L., Zheng, J., Whitlon, D. S., Garcia-Anoveros, J. & Bartles, J. R. Targeting of the hair cell proteins cadherin 23, harmonin, myosin XVa, espin, and prestin in an epithelial cell model. *J Neurosci* **30**, 7187-7201 (2010).
33 Hasson, T. & Mooseker, M. S. Porcine myosin-VI: characterization of a new mammalian unconventional myosin. *J Cell Biol* **127**, 425-440 (1994).
